# Chromatin dynamics of the *Klf4* locus in mouse pluripotent cells

**DOI:** 10.1038/s41598-026-45230-9

**Published:** 2026-03-27

**Authors:** Jente van Staalduinen, Hélène Kabbech, Selçuk Yavuz, Ridvan Cetin, Agnese Loda, Wiggert van Cappellen, Adriaan Houtsmuller, Kerstin Wendt, Ihor Smal, Frank Grosveld

**Affiliations:** 1 Former Department of Cell Biology, Erasmus University Medical Center, 3015 GE Rotterdam, The Netherlands; 2 Genome Biology and Epigenetics, Institute of Biodynamics and Biocomplexity, Department of Biology , Utrecht University, Utrecht, The Netherlands; 3 Department of Pathology, Erasmus University Medical Center, Rotterdam, The Netherlands; 4 Directors’ Research, European Molecular Biology Laboratory, Heidelberg, Germany; 5 Erasmus Optical Imaging Centre, Erasmus University Medical Center, Rotterdam, The Netherlands; 6 Cell Biology, Neurobiology and Biophysics, Department of Biology , Utrecht University, Utrecht, The Netherlands

**Keywords:** Single locus tracking, Chromatin dynamics, ANCHOR DNA labeling, *Klf4*, *Rad23b*, Cell biology, Computational biology and bioinformatics, Developmental biology, Genetics, Molecular biology

## Abstract

Understanding the factors involved in chromatin dynamics is crucial for the study of biochemical processes in which distant genomic regions need to come in close proximity. Previous single locus tracking studies suggest that chromatin dynamics are linked to active transcription, but studies which compare the chromatin dynamics between different locations within a defined chromatin domain are still very limited. Here we used the ANCHOR3 DNA labeling system to track multiple *cis*-regulatory elements and non-regulatory control regions at different positions in the mouse *Klf4* locus. We observe homogeneous chromatin motion of *cis*-regulatory elements and non-regulatory control regions in *Klf4* transcribing mESCs and their non-transcribing EpiLCs daughter cells. These observations challenge the notion that active transcription has a major effect on the locus dynamics of mammalian genes.

**Supplementary Information:**

The online version contains supplementary material available at 10.1038/s41598-026-45230-9.

## Introduction

Studies which track the movement of single genomic loci have led to different insights into the nature of chromatin dynamics. It has been shown to vary substantially between different nuclear locations^1^ and within the same topologically associated compartments (TADs)^2^. This heterogeneity in chromatin mobility suggests that local molecular processes, such as transcription and loop extrusion or chromatin state can influence the local mobility of chromatin. Many biochemical processes that take place on chromatin, are the result of distal genomic regions meeting in three dimensional space, hence understanding the dynamics of chromatin at single locus resolution is crucial.

Transcription has been associated with local confinement of some mammalian genes^3–6^, but not others^4,5^. Additionally, enhancers and promoters in transcriptionally active cells were shown to move in a fast-diffusing state^7^. The authors of that study introduced the stirring model which postulates that active transcription could stir chromatin in its local domain, leading to increased mobility of *cis*-regulatory elements and increased frequency of enhancer-promoter interactions.

Here, we test the prediction that local transcriptional activity leads to alterations in the chromatin dynamics of genomic regions. We perform these tests using *Klf4* as a model locus and the naïve-to-primed transition as a biological system. In naïve mouse embryonic stem cells (mESCs), 90% of the expression of *Klf4* is regulated by a locus control region (LCR)^8^ or now called a superenhancer^9^ ~ 55 kb downstream of the gene^10^. The *Klf4* enhancers and promoter have been shown to undergo chromatin looping^10,11^ and the frequency of their encounters is rate-limiting for transcription^12^. During differentiation to epiblast-like cells (EpiLCs), the enhancer becomes methylated^13^ and Klf4 expression is downregulated within 48 hours^14^. We use the ANCHOR3 DNA labeling system^15^ to track the movement of several genomic regions of the *Klf4* locus in mESCs and their derivative EpiLCs. The ANCHOR3 DNA labeling system uses the ParB/ParS components from a bacterial ParABS partitioning system. Upon sequence-specific recognition of ParS sites within the ANCH3 array sequence, OR3 (ParB) molecules fused to fluorophores oligomerize and spread along adjacent chromatin. This local recruitment of fluorophores enables live cell imaging of chromatin dynamics at single locus resolution.

We observe similar chromatin dynamics in *Klf4* transcribing mESCs and non-transcribing EpiLCs with the active *Klf4* promoter and enhancers showing confinement and diffusion coefficients similar to two local control regions at the time scale studied. Interestingly, we do observe faster movement of the promoter of the neighboring housekeeping gene *Rad23b* when compared to the *Klf4* promoter, enhancers or control regions. We propose that the local confinement of *cis*-regulatory elements upon transcriptional activation is not a general consequence of transcriptional activation and that local alterations in gene mobility upon transcriptional activation depend on chromatin context.

## Results

### Engineering of trackable cell lines

**Fig. 1:**
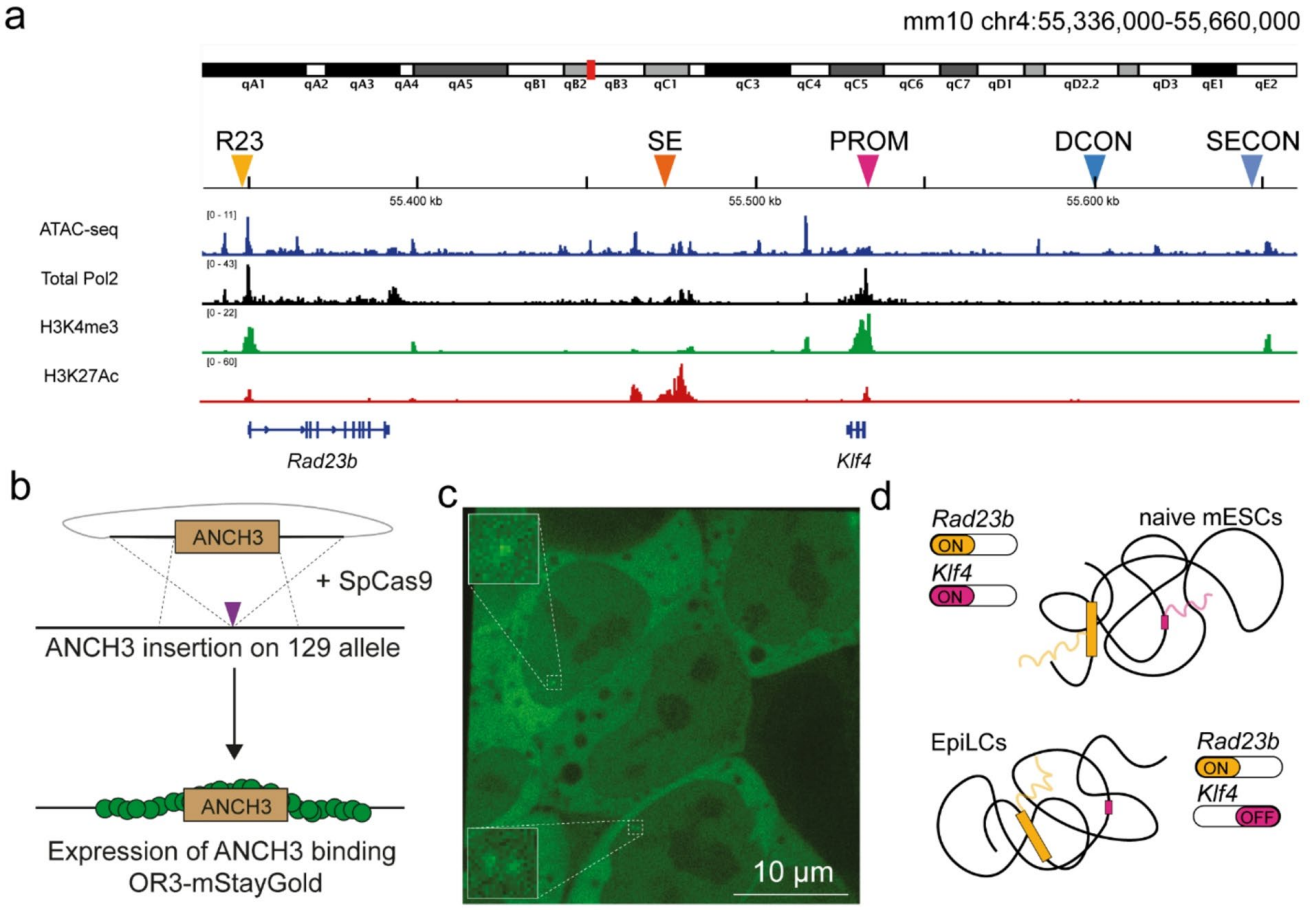
Endogenous labeling and live imaging of the *Klf4* locus. (**a**) Overview of the insertion positions for the imaging arrays in and around the *Klf4* locus as shown in the IGV genome browser together with the presence of open chromatin (ATAC-seq), total RNApol2 and active histone marks H3K4me3 and H3K27Ac in mESCs. Data were taken from the following publicly available datasets: ATAC-sequencing data of F121.6 mESCs cultured in serum/LIF conditions was downloaded from the 4D Nucleome Data Portal (4DNFI6HY3NE7)^20^. Chromatin immunoprecipitation coupled to sequencing data (ChIP-seq) of total Pol2 (GSM7187836)^21^, H3K4me3 (ENCFF240MDV)^22^ and H3K27Ac (ENCFF163HEV)^23^ were all from E14 mESCs cultured in serum/LIF conditions. (**b**) Workflow to implement ANCHOR3 DNA labeling. We used CRISPR/Cas9-mediated homology-directed insertion of ANCH3 arrays followed by expression of OR3-mStayGold with the PiggyBac transposon system. (**c**) Example image obtained from ANCHOR3 labeled *Klf4* promoters in mESCs with a close-up on diffraction limited spots representing a singlet and a doublet. (**d**) Our biological model system is the naïve-to-primed transition during which *Klf4* transcription is downregulated, while housekeeping gene *Rad23b* stays transcriptionally active.

We used CRISPR/Cas9 to introduce the ANCH3 array^15^ at five different locations along the *Klf4* locus (Fig. 1a). We inserted the ~ 1 kb imaging array at 1.5 kb downstream of enhancer 2 within the *Klf4* superenhancer (SE) and 2 kb from the transcription start site of *Klf4* and the neighbouring housekeeping gene *Rad23b* (Fig. S1). As additional controls, we introduced the array at two genomic locations in the gene desert upstream of the *Klf4* gene without active enhancer mark H3K27Ac or RNApol2 (Fig. 1a). One at a comparable distance from the promoter as the superenhancer region ~ 70 kb (distance control or DCON) and the other control region at ~ 117 kb upstream of the *Klf4* promoter (second control or SECON) (Fig. S2). All genome editing was performed in the hybrid F121.6 mESC line making use of SNPs in the 129 (129/Sv) and Castaneus (Cast/EiJ) allele to exclusively insert the ANCH3 array in the 129 allele (Fig. 1b, S1 and S2). These heterozygous cell lines were transfected with hyperactive PiggyBac transposase^16^ and a cargo containing the OR3 transgene directly fused to monomeric StayGold [QC2-6 FIQ variant]^17,18^ at its C-terminus and an antibiotic selection gene separated by an internal ribosomal entry site (Fig. 1b and S3). Stably selected cells were checked for OR3-mStayGold expression with flow cytometry. This showed that more than 95% of individual cells were OR3-mStayGold positive and that the levels of transgene expression between individual ANCH3 clones was comparable (Fig. S3). The fluorescence in these cell lines resembled the known subcellular distribution of OR3-eGFP fluorescence^3^. While most OR3-mStayGold proteins reside in the cytoplasm, a sufficient amount of OR3 proteins is present in the nucleus to form diffraction-limited spots at the genomic region of interest (Fig. 1c). To compare the motion of the *Klf4 cis*-regulatory elements between a transcriptionally active and inactive state, we used the naïve-to-primed transition as a biological model. Whereas both *Klf4* and *Rad23b* are transcriptionally active in naïve mESCs, only *Rad23b* remains transcriptionally active after differentiation to EpiLCs (Fig. 1d).

### Testing the invasiveness of ANCHOR3 DNA labeling

The levels of *Klf4* and *Rad23b* transcripts remained comparable after insertion and binding of the arrays (Fig. S4) (For one-way ANOVA results, see Table S7). Since the total transcription levels of the pluripotency factor *Klf4* might fluctuate between monoclonal cell lines and/or culture conditions, we also performed a pyrosequencing experiment on cDNA obtained from targeted cell lines. Using pyrosequencing, we can quantify the ratio between nascent transcripts from our targeted 129 allele and the clone-intrinsic reference Castaneus allele. We did not detect any consistent reduction in 129-specific *Klf4* nascent transcripts relative to Castaneus (Fig. S4). These control experiments show that we can visualize all five individual genomic regions in live cells without major perturbations to local transcription.

We next checked whether in our naïve culture condition all cells express Klf4 by performing an immunofluorescence staining for Klf4 and naïve marker Esrrb, to to ensure we can compare the movement of the *Klf4* superenhancer and *Klf4* promoter between transcriptionally active and inactive states. We find that the vast majority of cells are Klf4 and Esrrb double positive (Fig. S5). We then checked the efficiency of the differentiation to EpiLCs by RT-qPCR with a panel of markers for naïve mESCs and EpiLCs^19^. We see a consistent decrease in the naïve markers *Tbx3*, *Esrrb* and *Klf4* and upregulation of primitive markers *Dnmt3b* and *Fgf5* after differentiation to EpiLCs. In contrast to *Klf4*, the levels of housekeeping gene *Rad23b* did not change as a consequence of differentiation (Fig. S6). Immunofluorescence staining of Klf4 and primitive marker Oct6 in EpiLCs showed that on a single-cell level Klf4 protein expression is absent, while most cells show the presence of primitive marker Oct6 (Fig. S7).

### Technical specifications of locus tracking

We performed single locus tracking on the five different genomic regions in naïve mESCs and EpiLCs. We located diffraction-limited spots in a focal plane and performed 2D acquisitions on a spinning disk confocal microscope with 100 ms time intervals for a total duration of 1000 frames. Spots which show visual signs of doublets were discarded from further analysis (e.g. bottom close-up image in Fig. 1c). Timelapses were analysed using our tracking pipeline, which starts with the TrackMate plugin^24^ to detect spots and create tracks (Fig. S8) followed by a custom-made ImageJ/Fiji plugin^25^ which performs 2D Gaussian fitting to improve the localization accuracy of the spots initialized using coordinates obtained with TrackMate (Fig. S8). The cumulative exposure had limited effects on the mean spot intensity indicating that OR3-mStayGold spots do not lose much intensity due to photobleaching during our imaging window (Fig. S8), which can be attributed to the high photostability of mStayGold^18^, possibly in combination with turnover of individual OR3 molecules on the array^3^. We were able to track the majority of spots along the entire length of the video. The median track length was 989 frames in both naïve mESCs and EpiLCs and did not vary substantially between individual genomic regions (Fig. S9).

### Chromatin motion analysis

Tracks were analysed with a custom-made Python script (Fig. S10) which estimates two biophysical parameters from the mean square displacement (MSD) of individual tracks (time-averaged; TA-MSD) and collections of tracks (ensemble time-averaged; ETA-MSD): the diffusion coefficient (D) and the anomalous exponent (α). Within the framework of fractional Brownian motion (fBM), the extracted parameters α and D describe the diffusive behaviour of chromatin, where the MSD scales with time $MSD\left(\Delta t\right)\sim 2nD\Delta {t}^{\alpha }$. The diffusion coefficient reflects the effective mobility of a genomic region in its local environment, while the anomalous exponent quantifies deviations from normal diffusion. Subdiffusive behaviour (α < 1) is typically associated with confinement or viscoelastic constraints, whereas superdiffusive behaviour (α > 1) often arises from directed or active transport.

Comparison between the ensemble time-averaged MSD (ETA-MSD) of spots in live cells versus formaldehyde fixed cells showed that fixed spots, as expected, exhibit severely reduced mobility compared to spots in live cells (α = 0.07 for fixed spots, α = 0.48 for live cell spots) (Fig. S11). The movement of detected spots showed a negative dip in the velocity autocorrelation curve at short time lags as expected for chromosomal loci undergoing fBM^26^ (Fig. S11). Next, we plotted the cumulative distribution of XY displacements computed between consecutive frames, and we observed that the displacements of our genomic loci did not follow a self-similar Gaussian distribution as predicted for fBM^2^ (Fig. S11). This non-Gaussian distribution of displacements has been previously observed in tracking experiments of individual nucleosomes^27^.

**Fig. 2:**
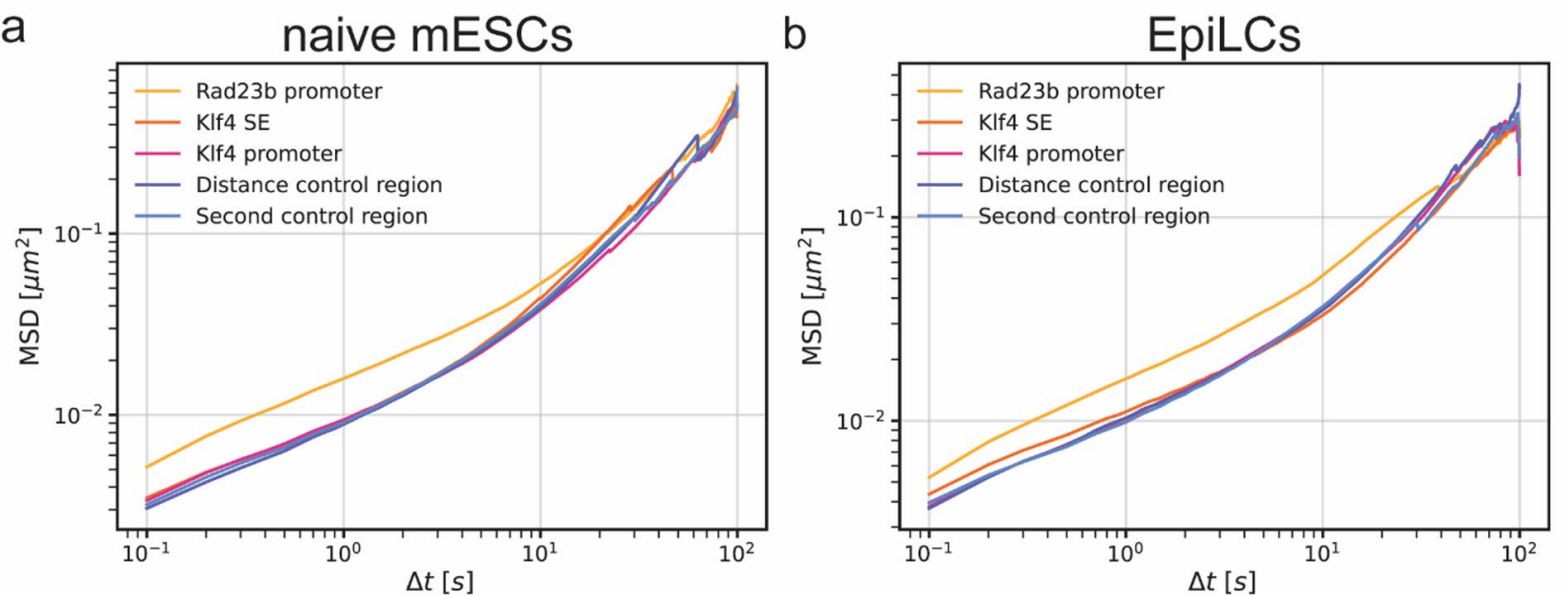
Ensemble time-averaged MSD curves of genomic regions in naive mESCs and EpiLCs. The ensemble time averaged mean squared displacement (ETA-MSD) curves of the different viewpoints of the *Klf4* locus in naive mouse embryonic stem cells (**a**) and epiblast-like cells (**b**).

**Fig. 3:**
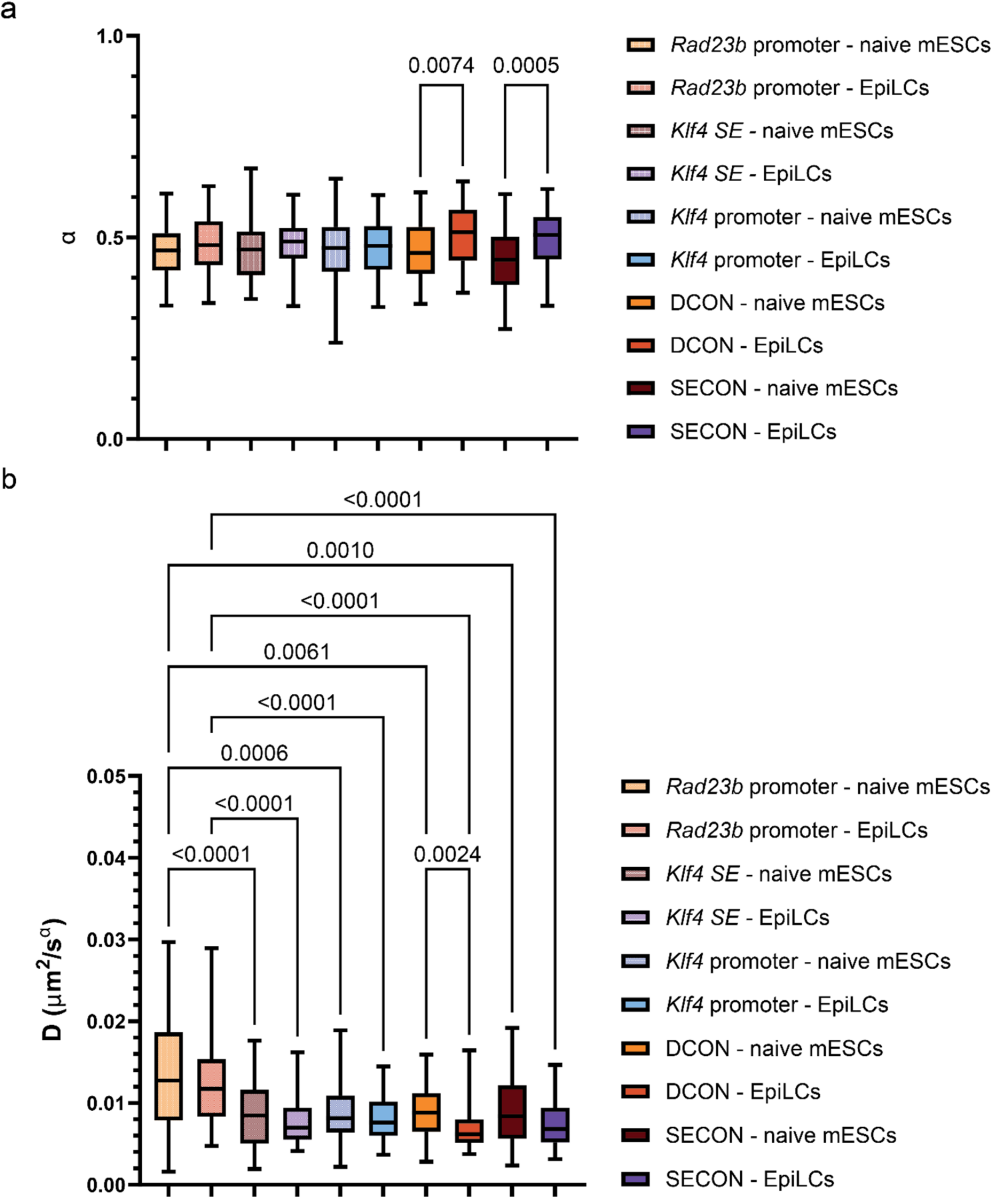
Motion parameters of genomic regions in naive mESCs and EpiLCs. Boxplots of the anomalous exponents (**a**) and diffusion coefficients (**b**) of genomic regions in naive mouse embryonic stem cells and epiblast-like cells. The boxes cover data from the interquartile range (IQR) with whiskers extending to the 5-95th percentile. Comparisons were made between the motion parameters of different genomic regions within a cell type and for every genomic region between the two cell types by performing the Kruskal–Wallis test followed by Dunn’s multiple comparisons test. The motion parameters were obtained from fitting the time-averaged MSD curves of tracking data obtained from three biological replicates, except two biological replicates for the distance control region in EpiLCs and two for the *Klf4* superenhancer in naive mESCs and EpiLCs.

Next, we obtained the anomalous exponent and diffusion coefficients from the time-averaged mean squared displacement (TA-MSD) curves of individual tracks (Fig. 3 and S12 and S13, see Table S8 for a summary of descriptive statistics). The anomalous exponents of the five genomic regions are very similar (Fig. 3a, Table S8) in naïve mESCs and EpiLCs. Moreover, the median values agree well with other locus tracking studies performed in the seconds-to-minutes range^28^. We did not observe significant differences between the anomalous exponents of the *cis*-regulatory elements (*Rad23b* promoter, *Klf4* superenhancer and *Klf4* promoter) versus the non-regulatory regions (DCON, SECON) within naïve mESCs or EpiLCs (Kruskal–Wallis test followed by Dunn’s multiple comparisons test) (Table S9). We can directly compare the diffusion coefficients of the five genomic regions in naïve mESCs and EpiLCs, firstly because the diffusion coefficient scales with the anomalous exponent and secondly because the anomalous exponents are comparable between the five genomic regions within a given cell type. This shows that the diffusion coefficient of the *Rad23b* promoter is significantly higher than that of all other genomic regions in both naïve mESCs and EpiLCs (Kruskal–Wallis test followed by Dunn’s multiple comparisons test) (Fig. 3b, Table S9). These differences can also be seen in the ETA-MSD curves of both naïve mESCs and EpiLCs where the motion of the *Rad23b* promoter stands out (Fig. 2—orange curves). It is proposed in the stirring model^7^, that the diffusion coefficient of transcriptionally active genomic regions should show a bimodal pattern with a slow and fast diffusing state. We tested the distribution of anomalous exponents and diffusion coefficients obtained from TA-MSDs for multi-modality (Hartigan’s dip test) and did not find statistical evidence of multi-modality for any of the genomic regions and conditions (Table S10).

The anomalous exponent and diffusion coefficients of genomic loci have previously been shown to differ between cell types^2,4,6,7^. We see a significant increase in the anomalous exponent of the distance control and second control region, and a decrease in the diffusion coefficient of the distance control after the naïve-to-primed transition (Kruskal–Wallis test followed by Dunn’s multiple comparisons test) (Fig. 3 and S14). This points to changes in the local chromatin dynamics unrelated to alterations in the local chromatin state. Despite downregulation of *Klf4* expression in EpiLCs, no significant differences were observed in the motion of the *Klf4* promoter or the *Klf4* superenhancer in the two cell types. The motion of the *Rad23b* promoter which does not alter transcriptional activity also did not change significantly. These observations highlight the benefit of probing multiple local viewpoints within one cell type to obtain a more complete picture of the relationship between local chromatin state and mobility.

We observed that the Rad23b promotermoves significantly faster than other genomic regions both in naïve mESCs and EpiLCs. Moreover, we detected a significant increase of the anomalous exponent and decrease of the diffusion coefficient of the distance control region after differentiation (Kruskal–Wallis test followed by Dunn’s multiple comparisons test) (Fig. S15, Table S8 for a summary of descriptive statistics; Table S9 for statistical analysis). These results show that cellular and nuclear motion do not significantly impact our ability to detect differences in chromatin dynamics between genomic regions and across cell types. movement or rotation of the cell or the nucleus in a subset of the tracking videos. Although we fit the MSD curves over short–lag-times (up to a maximum of 10% of the total track duration), during which the influence of such motion is limited, we performed an additional analysis to rule out its effect on the estimation of the diffusion parameters. We performed a principal component analysis (PCA) for the coordinates of each track and used the coordinates projected to the “short” axis representing the lowest variance (perpendicular to the “longest” axis that most likely represents substrate motion) to calculate 1D diffusion^29^ (Fig. S8). This alternative method of analysis confirms the observations in Figs. 2 and 3. We observed that the *Rad23b* promoter moves.

## Discussion

In this study, we quantified the chromatin dynamics of five locations along the *Klf4* locus. All locations show sub-diffusive chromatin dynamics in naïve mESCs and EpiLCs. Chromatin motion analyses suggested that active transcription does not have a major role in shaping the chromatin dynamics of the *cis*-regulatory elements of the *Klf4* locus. This is based on the observations that (1) the *Klf4* superenhancer and promoter do not show differences in the anomalous exponents or diffusion coefficients compared to two local control regions and (2) the mobility of the *Klf4* promoter or superenhancer does not significantly change upon loss of transcriptional activity during the naïve-to-primed transition.

Our observations that the motion of the active *Klf4* superenhancer and promoter does not differ from control regions are in stark contrast with a recent live cell imaging study of the *Sox2* locus^6^. Here, the authors find that the motion of the *Sox2* promoter and enhancer cluster are on average more confined than neighbouring chromatin. Since mammalian genes undergo transcriptional bursting with short bursts of transcriptional activity followed by periods of inactivity^30^, most tracking data is derived from poised alleles. Using the MS2-MCP live RNA labeling system, they show that for the *Sox2* promoter, the confinement was present on both transcribing and poised alleles, whereas the *Sox2* enhancer region was only confined on transcribing alleles. These data show that transcription-associated patterns in chromatin mobility can show up in motion parameters obtained at the population level. The homogenous motion of the *Klf4* superenhancer, promoter and the proximal control regions in our study therefore suggest that active transcription does not drastically alter the mobility of the *Klf4* superenhancer and promoter. The difference in the behaviour of the *Sox2* and *Klf4* loci is in line with other studies which show confinement of some genes during active transcription^3,5,6,31,32^, but not others^4,5,31,32^. What the differences are between these loci is as yet unknown.

A previous study from Gu et al. proposed the stirring model in which active transcription leads to increased mobility of *cis*-regulatory elements. These fast-diffusing states show up as a distinct population in the distribution of diffusion coefficients obtained from fitting individual tracks^7^. None of our tested genomic regions (*cis*-regulatory elements and non-regulatory control regions) showed statistical evidence of multi-modal distributions for the anomalous exponent and/or diffusion coefficient. For the *Sox2* locus, the enhancer and promoter also did not show multimodal distributions of motion parameters, while their motion parameters significantly differed between actively transcribing and poised alleles^6^. This suggests that the detection of multiple mobility states relies heavily on the locus and/or the statistical method of choice. We, therefore, cannot rule out the possibility that the *Klf4* superenhancer and promoter display subtle, but altered motion during transcriptional bursting, not picked up by the evaluation of the motion parameters from population data.

We also compared the movement of the specific chromatin locations between two cell types. The absence of significant changes in the motion of the *Klf4* promoter and superenhancer between naïve mESCs and EpiLCs suggests that their motion is unlikely to be significantly impacted by the transcription cycle. Instead we detect a significant decrease in the diffusion coefficient and increase in the anomalous exponent for the distance control region and subtle changes in the motion parameters of multiple genomic locations upon differentiation. These results underline the difficulty of drawing conclusions from directly comparing the mobility of an individual genomic region between multiple cell types. Cell-type specific changes in chromatin architecture and dynamics, such as domain-wide changes in cohesin loading could affect chromatin mobility throughout the locus rather than any individual genomic region. In addition, locus tracking results can be affected by differences in the efficiency of locus labeling between cell types.

The neighbouring *Rad23b* promoter shows significantly faster mobility than all *Klf4* viewpoints in both mESCs and EpiLCs. This observation verifies that chromatin dynamics can be very heterogenous within short genomic intervals (± 300 kb genomic region for this study)^2,6^. Moreover, it shows that we are able to pick up significant differences between the motion parameters of individual genomic regions. Most, if not all, mammalian genes show patterns of transcriptional bursting^33–35^. Therefore, it would be interesting to compare the chromatin dynamics between transcriptionally active and poised alleles of a housekeeping gene such as *Rad23b*. Housekeeping genes might serve as excellent model loci in which the effects of transcription on chromatin dynamics can be studied in isolation of other confounding factors, such as enhancer-promoter looping.

Cohesin-mediated loop extrusion has been shown to promote *Klf4* enhancer-promoter looping^12^ and *Klf4* is one of the few genes which is downregulated upon rapid depletion of Rad21^36^ and the cohesin release factor Wapl^36,37^ in mouse embryonic stem cells. The *Klf4* locus is therefore an interesting model locus for studying the effect of loop extrusion on enhancer-promoter looping in live cells. Moreover, live labeling of the *Klf4* superenhancer, which has been genetically dissected in mouse embryonic stem cells before^10,14,38^, could serve as a promising tool to study the molecular assembly of an active chromatin hub near the *Klf4* gene^12^ in live cells. We anticipate that the mouse *Klf4* locus will be a valuable addition to model loci in which the interplay between local chromatin dynamics, loop extrusion and transcription can be studied in live cells.

### Limitations of this study

In this study, we use allele-specific genome editing to exclusively create trackable locations on the 129 chromosome, while using the Castaneus allele as a clone-intrinsic internal reference. The downside of this approach is the reliance on a single available spot per cell for locus tracking. During our imaging window (100 s), movement of the cell or nucleus prohibits us from studying the motion of these arrays in the range of minutes. In future studies, locus trajectories could be corrected by measuring the changes in radial distance between two orthogonal labels on a single chromosome or homozygous integrations of a single label. This will enable the measurement of chromatin dynamics of the *Klf4* locus in the minutes to hours range plus in three dimensions of space.

## Methods

### Cell culture

#### Serum/LIF condition

F1-21.6 mESCs (Courtesy of Joost Gribnau) were cultured in serum/LIF conditions consisting of HyClone DMEM (Cytiva, Catalog no: SH30081.01), 15% Fetal Bovine Serum (Capricorn, Catalog no: FBS-12A, Lot no: CP18-2152 and Lot no: CP18-2112), 1X GlutaMAX (Gibco, Catalog no: 11140050), 1 × MEM Non-Essential Amino Acids (Gibco, Catalog no: 11140050), 10 mM HEPES (Cytiva, Catalog No: SH30237.01), 0.1 mM 2-Mercaptoethanol (Gibco, Catalog no: 31350010), 1% Penicillin/Streptomycin (Gibco, Catalog no: 15140122) and 1,000 units/mL Leukemia Inhibitory Factor (LIF) (ESGRO, Catalog no: ESG1107). Cell culture dishes were coated with 0.2% gelatin (Sigma-Aldrich, Catalog no: 04055-500G) and pre-seeded with a monolayer of irradiated mouse embryonic fibroblasts (MEFs) (mouse strain C57BL/6, Erasmus Medical Center iPS Core Facility). Medium was refreshed daily and cells were passaged every other day using Trypsin–EDTA (Sigma-Aldrich, Catalog No: T3924) and seeded at a density of 1,5 × 10^4^ cells/cm^2^. Cells were maintained in a humidified incubator at 37 °C and 5%CO_2_. Cell lines in serum/LIF conditions were frozen down using cell culture medium supplemented with 10% DMSO.

#### Naïve condition

In naive conditions, cell lines were cultured in N2B27 medium consisting of a 1:1 mixture of Neurobasal medium (Gibco, Catalog no: 21103049) and DMEM/F-12 supplemented with GlutaMAX (Gibco, Catalog no: 31331093), 0.5X GlutaMAX (Gibco, Catalog no: 11140050), 0.5X N-2 Supplement (Gibco, Catalog no: 17502048), 0.5X B-27 Supplement minus vitamin A (Gibco, Catalog no: 12587010), 7.5% solution Bovine Albumin Fraction V (Gibco, Catalog no: 15260037, Lot no: 2229672), 50 µM 2-Mercaptoethanol (Gibco, Catalog no: 31350010) supplemented with 3 µM CHIR 99,021 (Tocris Bioscience, Catalog no: 4423/50), 1 µM PD0325901 (Selleck chem, Catalog no: S1036) and 1,000 units/mL Leukemia Inhibitory Factor (LIF) (ESGRO, Catalog no: ESG1107). When culturing the OR3-mStayGold stably selected cell lines, 200 µg/mL Geneticin (Gibco, Catalog no: 10131035) was added to the N2B27 2i/LIF medium to maintain transgene expression. Before passaging, cell culture plates were coated with Geltrex (Gibco, Catalog no: A1413302) for 1 h at 37 °C. Cells were passaged every other day by detaching the cells using Accutase (Millipore, Catalog no: SCR005) treatment for 6 min at room temperature. The cell suspension was washed in a DMEM/F-12 (Gibco, Catalog no: 31331093) solution with 0,11% Bovine Albumin Fraction V (Gibco, Catalog no: 15260037), counted and seeded at a density of 1,5 × 10^4^ cells/cm^2^ as described previously^39^. Cells were maintained in a humidified incubator at 37 °C and 5%CO_2_. Freezing of cell lines in the naïve culture condition was performed by resuspending the cells in the serum-free CryoStor cell cryopreservation media (Sigma Aldrich, Catalog No: C2874-100ML). All cell lines were routinely checked for mycoplasma contamination and tested negative.

#### Differentiation to EpiLCs

12 well plates or 8 Well Glass Bottom µ-slides (ibidi, Catalog no: 80827) were coated with Geltrex (Gibco, Catalog no: A1413302) for 1 h at 37 °C. Cells cultured in N2B27 2i/LIF conditions were obtained using Accutase (Millipore, Catalog no: SCR005) treatment for 6 min at room temperature, washed and resuspended in differentiation medium (N2B27 imaging medium (see **Cell culture** and **Live cell imaging**), 3 ng/mL Activin A (PeproTech, Catalog no: 120–14), 2 µM porcupine inhibitor IWP2 (Selleck chem, Catalog no: S7085-10 mg), 1 µM inverse pan-retinoic acid receptor (RAR) agonist BMS493 (MedChemExpress, Catalog no: HY-108529) and 10 µM ROCK inhibitor Y-27632 (STEMCELL Technologies, Catalog no: 72304) (conditions adapted from^40^). Cells were plated at a density of 2,5 × 10^4^ cells/cm^2^ and allowed to differentiate for 2 days.

### Plasmid cloning

All cloning reactions were carried out with 25 μl of NEB 5-alpha Competent E. coli (New England Biolabs, Catalog no: C2987H) using heat-shock at 42 °C. Transformants were picked and plasmid DNA was purified with the QIAprep Spin Miniprep Kit (QIAGEN, Catalog no: 27106) and/or the NucleoBond Xtra Midi kit (MACHEREY–NAGEL, Catalog no: 740410.50).

#### SpCas9 cloning

The CRISPR/Cas9-mediated insertions of the ANCH3 sequence were performed using SpCas9 and the SpCas9 HF1 variant (N497A; R661A; Q695A; Q926A). pSpCas9(BB)-2A-Puro (PX459) V2.0 was a gift by Feng Zhang (Addgene plasmid #62,988; http://n2t.net/addgene:62988; RRID:Addgene_62988)^41^ and the B-SpCas9-HF1 plasmid was a gift by Ervin Welker (Addgene plasmid #126,762; http://n2t.net/addgene:126762; RRID:Addgene_126762)^42^. In the B-SpCas9-HF1 plasmid, the amino acids 1005–1013 are replaced with two glycine to ensure efficient cleavage of 5’ extended 21 nucleotide spacer sequences. The B-SpCas9-HF1 was modified to incorporate the U6 promoter and 2A PuroR sequence from the PX459 V2.0 plasmid [FseI (5499)—KpnI (438)]. This was achieved via a restriction-ligation cloning using FseI (New England Biolabs, Catalog no: R0588L) and KpnI-HF (New England Biolabs, Catalog no: R3142S). This new plasmid is designated as B-SpCas9-HF1-2A-PuroR.

#### Single guide RNA cloning

Single guideRNAs for the insertion of the ANCH3 sequence in the 129 allele were designed with the help of web tool CRISPOR version 4.98^43^. Allele-specific guideRNAs were identified by the build-in CRISPOR variant database and verified by checking the 129S1_SvImJ and CAST_EiJ UCSC assemblies. In case the target sequence did not start with a guanine, a 5′ G was added to the target sequence to ensure efficient expression from the U6 promoter. Single stranded oligos containing the target sequence with a 5’ CACC overhang and the reverse complement of the target sequence with a 5’ AAAC overhang were annealed in 1X NEB Buffer 2.1 (New England Biolabs, Catalog no: B7202S) by heating up the sample to 95 °C for 5 min and ramping down the temperature to 25 °C by 5 °C per minute in a thermocycler (final concentration 10 µM). To insert the target sequence in the plasmid backbones, PX459 V2.0 and B-SpCas9-HF1-2A-PuroR were digested with 1 μl BbsI-HF (New England Biolabs, Catalog no: R3539L) at 37 °C O/N. The digested plasmid was purified via agarose gel electrophoresis followed by a spin column clean up using the Monarch DNA Gel Extraction Kit (New England Biolabs, Catalog no: T1020S) according to manufacturer’s instructions. 100 ng of the linearized plasmid and 2 μl of a dilution of the annealed duplex [40 nM] were ligated with 1 μl T4 DNA ligase (New England Biolabs, Catalog no: M0202S) O/N at 16 °C. Target sequence insertions were verified using Sanger sequencing. Single guideRNA target sequences and their respective plasmid backbones can be found in Table S1 and S2.

#### ANCH3 homology donor vectors

For the *Klf4* superenhancer, *Klf4* promoter and Distance control region, the homology donor sequence was amplified from 129X1/SvJ purified genomicDNA using the blunt-end Phusion High-Fidelity DNA Polymerase (New England Biolabs, Catalog no: M0530L). The PCR product was cloned in the pCR-Blunt II-TOPO vector according to the manufacturer’s instructions (Invitrogen, Catalog no: 450245). Hereafter, a cloning site containing an I-SceI and BglII recognition motif (5’ TAGGGATAACAGGGTAATAGATCT 3’) was inserted in the expected cleavage position (between 3 and 4 bp 5’ of the PAM site) via a Q5 Site-Directed Mutagenesis reaction (New England Biolabs, Catalog no: E0554S). For the *Rad23b* promoter and second control region, gBlocks Gene Fragments (synthesized by IDT, see Supplementary sequence 1 and 2 in Supplementary Material 1) containing homology arms (sequence from GCA_001624185.1, 129S1_SvImJ_v1 assembly) and the I-SceI/BglII cloning site were directly cloned into the pCR-Blunt II-TOPO vector using the Zero Blunt TOPO PCR Cloning Kit. In another round of Q5 Site-Directed Mutagenesis, a LoxP site was added facing the *Klf4* enhancers 1 and 2 (*Klf4* superenhancer), the *Klf4* gene body (*Klf4* promoter) and in the second control region (SECON) facing the gene desert. The original gBlocks Gene Fragment of the *Rad23b* promoter region already contained a LoxP site facing the *Rad23b* gene.

The homology donor entry vectors of all genomic regions were sequentially digested with BglII (New England Biolabs, Catalog no: R0144S) and I-SceI (New England Biolabs, Catalog no: R0694L). The final ANCH3 homology donor constructs were generated by ligation of the I-SceI and BamHI-HF (New England Biolabs, Catalog no: R3136S) digested ANCH3 fragment from the pcDNA5 FRT I-Sce1 ANCH3 plasmid (NeoVirTech) into the I-SceI/BglII linearized homology donor entry vectors using T4 DNA ligase (New England Biolabs, Catalog no: M0202S). An overview of the cloning reactions can be found in Table S3.

#### Transgene

The PiggyBac cargo entry plasmid consisted of a combination of the backbone [AflII (4477)—PspXI (1866)] of the epB_CAG_TetRFlag-nls-tdTom_DEx4 plasmid which was a gift by Orion Weiner (Addgene plasmid #119,909; http://n2t.net/addgene:119909; RRID:Addgene_119909)^44^ and the IRES2-NeoR-SV40 poly(A) signal sequence [3409—5054] amplified from the PB533_2E12LI-sfGFP plasmid which was a gift by Hiroshi Kimura (Addgene plasmid #167,527; http://n2t.net/addgene:167527; RRID:Addgene_167527)^45^. The OR3 cDNA sequence was amplified from the 267_9 peGFP-c1 OR3 plasmid (NeoVirTech) and mStayGold [QC2-6 FIQ]^18^ was amplified from pMP136 pGB mSG (kindly provided by Maarten Paul) using Phusion High-Fidelity DNA Polymerase (New England Biolabs, Catalog no: M0530L). The PiggyBac cargo entry plasmid was digested with PspXI (New England Biolabs, Catalog no: R0656S) and NotI-HF (New England Biolabs, Catalog no: R3189L) and purified via agarose gel electrophoresis followed by a spin column clean up using the Monarch DNA Gel Extraction Kit (New England Biolabs, Catalog no: T1020S) according to manufacturer’s instructions. The OR3 and mStayGold PCR products were inserted into the PiggyBac cargo entry plasmid via Gibson Assembly (New England Biolabs, Catalog no: M5510AA) following the manufacturer’s instructions. The sequence of the PiggyBac cargo entry backbone can be found in Supplementary sequence 3 in Supplementary Material 1.

### Generation of locus tracking cell line

#### Genome editing of ANCH3 knock-in cell lines

F1-21.6 mESCs were transfected with 1 µg of a sgRNA/SpCas9-2A-PuroR expression vector and 2 µg of a homology donor plasmid containing the ANCH3 sequence (see section **Plasmid cloning**) with Lipofectamine 2000 Reagent (Thermo Scientific, Catalog no: 11668019) according to manufacturer’s instructions (12 µL of Lipofectamine 2000 Reagent). After incubation of the plasmid DNA-lipid complexes in Opti-MEM Reduced-Serum Medium (Gibco, Catalog no: 51985026) at room temperature for 30 min, the reaction mix was combined with 600,000 cells diluted in Opti-MEM Reduced-Serum Medium and placed on a shaking platform (125 rpm) inside an incubator. After 15 min, 200,000 cells were added to a 10 cm dish containing pre-warmed serum/LIF medium and a monolayer of irradiated B6-Puro feeders (Gibco, Catalog no: A34965). After 24 h, medium was refreshed with serum/LIF medium containing 1 µg/mL puromycin (Sigma-Aldrich, Catalog no: P8833-10MG). After two days of antibiotic selection, the medium was refreshed with standard serum/LIF medium. Individual colonies were picked with the help of a microscope placed in a hood, trypsinized and divided into two wells of a 96-well plate pre-seeded with feeders at day 4–5 post transfection.

#### Genotyping of ANCH3 knock-in cell lines

96-well plates containing individual clones were washed with 200 µl PBS and incubated with 100 µl of genomic DNA lysis buffer (10 mM Tris–HCl pH 8.0, 10 mM EDTA, 0,08% SDS, 10 mM NaCl, RNAse A (Calbiochem, Catalog no: 55674–50) and Proteinase K (Roche, Catalog no: 3115852001) at 55 °C while shaking at 500 rpm for 1–4 h. This was followed by the addition of 100 µL of isopropanol to precipitate the genomic DNA. After two washes with 200 µL of 70% ethanol, pellets were air-dried in a dry incubator for 30 min to 1 h at 37 °C before resuspension in 50 µL of nuclease-free H_2_O. An overview of the genotyping primers and PCR reaction conditions can be found in Table S4.

#### Generation of OR3-mStayGold expressing cell lines

ANCH3 KI mESCs were transfected with 0.5 µg of an EF-1α HyPBase expression vector (gift by Alex Zelensky, Erasmus MC) and 2 µg of the PiggyBac pCAG OR3-mStayGold IRES2 NeoR cargo plasmid (see section **Plasmid cloning**) with Lipofectamine 2000 Reagent (Thermo Scientific, Catalog No: 11668019) according to manufacturer’s instructions (10 µL Lipofectamine 2000 Reagent). After incubation of the plasmid DNA-lipid complexes in Opti-MEM Reduced-Serum Medium (Gibco, Catalog no: 51985026) for 30 min at room temperature, the reaction mix was combined with 600,000 cells diluted in Opti-MEM Reduced-Serum Medium and placed on a shaking platform (125 rpm) inside an incubator. After 15 min, 200,000 cells were added to a 6 well plate containing pre-warmed serum/LIF medium and a monolayer of irradiated feeders. After 24 h, medium was refreshed with serum/LIF medium. Four days after transfection with the PiggyBac OR3-mStayGold cargo, cells were seeded on 0.2% gelatin coated plates in serum/LIF condition without MEFs. The day after, medium was switched to N2B27 2i/LIF medium (see **Cell culture**). The transfection pool was subsequently selected for transgene expression in N2B27 2i/LIF medium with 400 µg/mL Geneticin (Gibco, Catalog no: 10131035) for 4 days. This antibiotic treatment also resulted in the efficient removal of any residual feeders from the serum/LIF culture condition. Hereafter, cell lines were routinely cultured in N2B27 2i/LIF medium containing 200 µg/mL Geneticin to maintain transgene expression.

### Flow cytometry

In order to assess the levels of OR3-mStayGold expression in the transfection pools, cell lines stably selected for transgene expression were subjected to flow cytometry measurements. Cells cultured in naïve conditions were obtained from a 6 well plate via Accutase (Millipore, Catalog no: SCR005). The cell suspension was washed with wash buffer (see **Cell culture**) and moved through a 40 µm Cell Strainer (Falcon, Catalog no: 352340). After centrifugation, the cell pellet was resuspended in N2B27 2i/LIF medium and stored on ice until measurement. Measurements of fluorescent intensity in the BB 542/27 channel were performed on a FACSAria II SORP (BD Biosciences). Flow cytometric data analysis was subsequently performed with FlowJo (version 10.10.0).

### RNA expression analyses

RNA was extracted from stably selected transfection pools cultured on a 12 well plate in naïve and differentiation conditions with the ReliaPrep RNA Cell Miniprep system (Promega, Catalog no: Z6012) according to manufacturer’s instructions. After RNA isolation, RNA was treated with 1 μL TURBO DNase for 30 min at 37 °C (Invitrogen, Catalog no: AM1907). Hereafter, 1 µg of RNA was converted to cDNA using 1 μl of 50 µM Oligo d(T)_20_ (IDT, Catalog no: 51-01-15-01) or 50 µM random hexamers (IDT, Catalog no: 51-01-18-01) using the SuperScript IV Reverse Transcriptase kit according to manufacturer’s instructions (Invitrogen, Catalog no: 18090050). After the reverse transcription, the cDNA was diluted five times with TE buffer pH = 8.0.

#### RT-qPCR

The expression levels of the naïve marker *Tbx3*, the epiblast markers *Fgf5*, *Dnmt3b* and the target genes *Klf4* and *Rad23b* were assessed in a RT-qPCR reaction using 2 μl cDNA (Oligo dT-primed) input per well. The qPCR reaction was performed using Platinum Taq DNA Polymerase (Invitrogen, Catalog no: 10966034) and 0.15X intercalating dye SYBR Green I nucleic acid gel stain (Sigma Aldrich, Catalog no: S9430). The PCR was performed on a CFX96 Real-Time System using the following amplification protocol: initial denaturation 95 °C [2 min] followed by 40 PCR cycles of denaturation 95 °C [30 s], annealing 60 °C [30 s] + plate read and extension 72 °C [30 s]. After another denaturation step of 95 °C [10 s], a melt curve analysis was performed by increasing the temperature from 65 °C to 95 °C by an increment of 0.5 °C every 5 s followed by a plate read. The Cq values of *Klf4* and *Rad23b* were normalized against the levels of reference genes *Sdha* and *Tbp* and the values were compared to the expression levels of non-targeted clones ran on the same qPCR plate using the 2^-ΔΔCq^ method (Fig. S4). For the statistics,—ΔΔCq values were taken as input (Table S7). In order to check the efficiency of epiblast differentiation, the levels of naïve and primed expression markers were normalized against housekeeping genes Sdha and Tbp (2^-ΔCq^ method) and compared between naïve and primed conditions on the same plate (Fig. S6). The primer sequences used in the RT-qPCR reactions can be found in Table S5.

#### Pyrosequencing

The use of the hybrid F1-21.6 cell line allows for the internal comparison between *Klf4* transcripts of the 129 and Cast allele. To this end, 2 μl cDNA (primed with random hexamers) was subjected to PCR amplification using Platinum Taq DNA Polymerase (Invitrogen, Catalog no: 10966034) supplemented with 3% DMSO (Thermo Scientific, Catalog no: F-515). Since the forward primers contained a 5’ Biotin modification, the PCR product can be purified with streptavidin beads and the reaction can be run on a Pyrosequencing machine. Table S5 shows the primer sequences and PCR conditions used in the pyrosequencing experiment. PCR reactions performed on 20 ng/μl purified genomic DNA from the parental F1-21.6 cell line and a 129X1/SvJ cell line were used as reference samples.

### Immunofluorescence

Samples were washed twice with PBS and incubated with 4% formaldehyde in PBS (diluted from Pierce, Catalog no: 28906) for 10 min at room temperature. After a quick wash with PBS containing 0.05% TWEEN 20 (Sigma Aldrich, Catalog no: P1379-250ML) (PBS-T), cells were permeabilized in PBS containing 0.1 M Glycine (Sigma Aldrich, Catalog no: G7126) and 0.3% Triton X-100 (Sigma Aldrich, Catalog No: T8787) for 10 min. Following one more wash in PBS-T, samples were blocked for at least 1 h at room temperature in blocking buffer (PBS-T containing 1% BSA (Sigma Aldrich, Catalog no: A3294-100G) and 5% normal donkey serum (Jackson ImmunoResearch, Catalog no: 017–000-121). Hereafter, samples were incubated with the primary antibody diluted in blocking buffer at room temperature overnight. After three wash steps of 5–10 min with PBS-T, secondary antibodies diluted in blocking buffer were incubated for 2–3 h in the dark at room temperature. After three washes with PBS-T, samples were immersed in ProLong Gold antifade reagent with DAPI (Invitrogen, Catalog no: P36931) and allowed to cure overnight. A list of primary and secondary antibodies can be found in Table S6.

Images with a pixel size of 71 × 71 nm and 4 time line averaging, were produced by a Leica SP8 AOBS microscope with white light laser using a 40x/1.30 NA HC PL APO CS2 objective in combination with appropriate laser lines and emission filters (excitation with 405 nm excitation laser and a BP 420–460 nm emission filter for DAPI, 561 nm excitation laser and a BP 570–610 nm emission filter for Cy3 and 650 nm excitation laser and BP 670–800 nm emission filter for Alexa Fluor 647). A tile scan of 3 by 3 tiles with 10% overlap was recorded for each condition in order to image several hundred cells per condition. In total, 9 tile scans (acquired during 3 independent experiments) were recorded for each condition.

Quantitative image analysis was performed in order to calculate the average fluorescence intensity of Klf4 and Esrrb/Oct6 across all conditions with a homemade macro in the Fiji variant^25^ of ImageJ^46^. First, nuclear regions were segmented by using the DAPI signal and the StarDist 2D plugin^47^. Next, the mean intensity values of Klf4 and Esrrb or Oct6 inside the defined nuclear areas were measured.

### Live cell imaging

One day before imaging naïve cells, a µ-slide 8 Well Glass Bottom (ibidi, Catalog no: 80827) was coated with 5 µg/mL laminin 511 LN (BioLamina, Catalog no: LN511-0502) in DPBS containing calcium and magnesium (Gibco, Catalog no: 14040091) and incubated for 1–2 h at 37 °C. Hereafter, the coating solution was removed and 100,000 cells were seeded in N2B27 2i/LIF imaging medium (N2B27 2i/LIF with Neurobasal medium minus phenol red (Gibco, Catalog no: 12348017) and DMEM/F-12 minus phenol red (Gibco, Catalog no: 21041025)). At the day of imaging, medium was refreshed. For the imaging of epiblast-like cells, a µ-slide 8 Well Glass Bottom (ibidi, Catalog no: 80827) was coated with Geltrex (Gibco, Catalog no: A1413302) for at least one hour at 37 °C. Two days before imaging, 25,000 cells were seeded in differentiation imaging medium (see **Differentiation to epiblast-like cells (EpiLCs)** for details).

Stream acquisitions of 1,000 frames were acquired on a Ti-Eclipse inverted microscope (Nikon) with Spinning Disc unit CSU-X1 (Yokogawa) using a 100 × /1.49 NA oil immersion objective (pixel size = 67 nm) and the 49,002 ET—EGFP (Chroma) filter set with a 491 nm laser. Images were captured with the following settings: 100 ms exposure time and EM 10 MHz (100 V gain) on a QuantEM512C 512 × 512pixels 16bit (Photometrics) with the Perfect Focus System (Nikon) switched on. During imaging, cells were maintained under 5%CO_2_ and 37 °C conditions using a Tokai Hit incubator.

### Spot tracking

Tracking coordinates were obtained by TrackMate v7^24^ in Fiji^25^. Time lapses with visible sister chromatids were discarded from the analysis. Regions of interest (ROI) containing the diffraction limited spots were drawn and spots within the ROIs were detected using the Hessian detector (default normalized quality value = 0.7) or the Laplacian of Gaussian detector (default value = 25) with an estimated object diameter of 10 pixels with subpixel resolution enabled. The simple LAP tracker was applied with the following parameters: linking max distance = 5 pixels, maximum gap closing distance = 5 pixels and gap-closing max frame gap = 3. Only (sub)tracks which span a minimum of 50 frames were kept and tracks were manually inspected to ensure accuracy.

To refine the spot positions, the time lapses and tracking files exported from TrackMate were loaded in the custom-made Fiji plugin “GaussFit to TrackMate Tracks” (https://smal.ws/wp/gaussian-trackmate-localization/). A 9 × 9 pixel 2D Gaussian fitting was initialized at the TrackMate coordinates. The settings as defined by the initial user dialog were: Minimum spot intensity (above the background): 10.0, Maximum spot intensity (above the background): 65,000, Minimum stdev (sigma) of Gaussian PSF: 0.36, Maximum stdev (sigma) of Gaussian PSF: 3.60, Step in the spot intensity range (for greedy search): 1.000, Step in the PSF sigma range (for greedy search): 0.040 and Size of (n x n)-patch for Gaussian PSF fit, n: 9. If the Gaussian fitting did not result in a successful fit, the original TrackMate coordinate was taken for that time point. The plugin returned a tracking XML file and a table containing the fitting results, including the spot intensity per frame (used to plot Fig. S8).

The coordinates of the tracks also underwent a principal component analysis (PCA) representing each track in a new coordinate system, with “longest” and “shortest” axes. To calculate 1D PCA diffusion, the displacements projected onto the axis perpendicular to the longest axis were taken as input. The resulting tracking files (PCA.Gauss.tracks.xml) were taken as input for the alternative MSD analysis (Fig. S8).

### Chromatin motion analysis

Tracking files were first refined with the “GaussFit to TrackMate Tracks” plugin (Gauss.tracks.xml/PCA.Gauss.tracks.xml). Refined tracks obtained from individual spots were analyzed using a custom Python script, publicly available at https://github.com/hkabbech/spot-analysis. Coordinates $r\left( t \right) = \left( {x\left( t \right),y\left( t \right)} \right)$. from the tracks were used to compute the time-averaged MSD (TA-MSD) curves at $T$ discret times:

$$TA - MSD\left( \delta \right) = \frac{1}{T - \delta }\mathop \sum \limits_{i = 1}^{T - \delta } \left| {r\left( {t_{i} } \right) - r\left( {t_{i} + \delta } \right)} \right|^{2}$$

For the fitting of the TA-MSD curves, either 10% of the maximum track length was taken or at least 10 frames. These curves were fitted using the MSD power-law equation to extract the anomalous exponent α and diffusion coefficient D: $MSD\left(\Delta t\right)\sim 2nD\Delta {t}^{\alpha }$, with $n$ the number of dimensions. For each condition, $N$ tracks were combined to compute the ensemble time-averaged MSD (ETA-MSD) curves:

$$ETA - MSD\left( \delta \right) = \frac{1}{N}\mathop \sum \limits_{i = 1}^{N} TA - MSD_{i} \left( \delta \right)$$

The velocity autocorrelation curve and Gaussianity (Fig. S11) were also calculated based on the trajectory displacements.

## Supplementary Information

Below is the link to the electronic supplementary material.

Supplementary Material 1

Supplementary Material 2

## Data Availability

The ANCHOR3 DNA labeling system for locus visualization is licensed from NeoVirTech. The imaging datasets generated during the current study are available in the BioImage Archive ^48^ repository (http://www.ebi.ac.uk/bioimage-archive) under accession number S-BIAD2419. The code used for the chromatin motion analyses is freely available via GitHub page [https://github.com/hkabbech/spot-analysis] and the custom-made Fiji plugin can be accessed via [https://smal.ws/wp/gaussian-trackmate-localization/]. F1-21.6 ATAC-sequencing data used in **Fig. 1** , **S1** and **S2** were downloaded from the 4DN Data Portal (4DNFI6HY3NE7) ^20^. Publicly available ChIP-seq data for Polr2a (GSM7187836) were downloaded from Wang et al. ^21^ and H3K4me3 (ENCFF240MDV) ^22^ and H3K27Ac (ENCFF163HEV) ^23^ ChIP-seq data from the ENCODE Project were used in panel (a) from **Fig. 1** .
