# Supplementary material for: Chromatin dynamics of the Klf4 locus in mouse pluripotent cells
Source: Sci Rep. 2026 Mar 27;16:10941. doi: 10.1038/s41598-026-45230-9 (PMC13039108; doi:10.1038/s41598-026-45230-9)
Supplement: Supplementary file 2 — Supplementary Material 2 [file 41598_2026_45230_MOESM2_ESM.docx]

**Chromatin dynamics of the *Klf4* locus in mouse pluripotent cells**

Jente van Staalduinen^1,2^, Hélène Kabbech^1^, Selçuk Yavuz^3^, Ridvan Cetin^1^, Agnese Loda^4^, Wiggert van Cappellen^5^, Adriaan Houtsmuller^3,5^, Kerstin Wendt^1^, Ihor Smal^1,6^, Frank Grosveld^*1^

^1^ Former Department of Cell Biology, Erasmus University Medical Center, Rotterdam, The Netherlands

^2^ Genome Biology and Epigenetics, Institute of Biodynamics and Biocomplexity, Department of Biology, Utrecht University, Utrecht, The Netherlands

^3^ Department of Pathology, Erasmus University Medical Center, Rotterdam, The Netherlands

^4^ Directors' Research, European Molecular Biology Laboratory, Heidelberg, Germany

^5^ Erasmus Optical Imaging Center, Erasmus University Medical Center, Rotterdam, The Netherlands

^6^ Division of Cell Biology, Neurobiology and Biophysics, Department of Biology, Utrecht University, Utrecht, The Netherlands

* Corresponding author:

Frank Grosveld, Department of Cell Biology, Erasmus University Medical Center, 3015 GE Rotterdam, The Netherlands, e-mail: f.grosveld@erasmusmc.nl

**Supplementary material 1**

Supplementary figures

Supplementary sequence 1

Supplementary sequence 2

Supplementary sequence 3

References

**
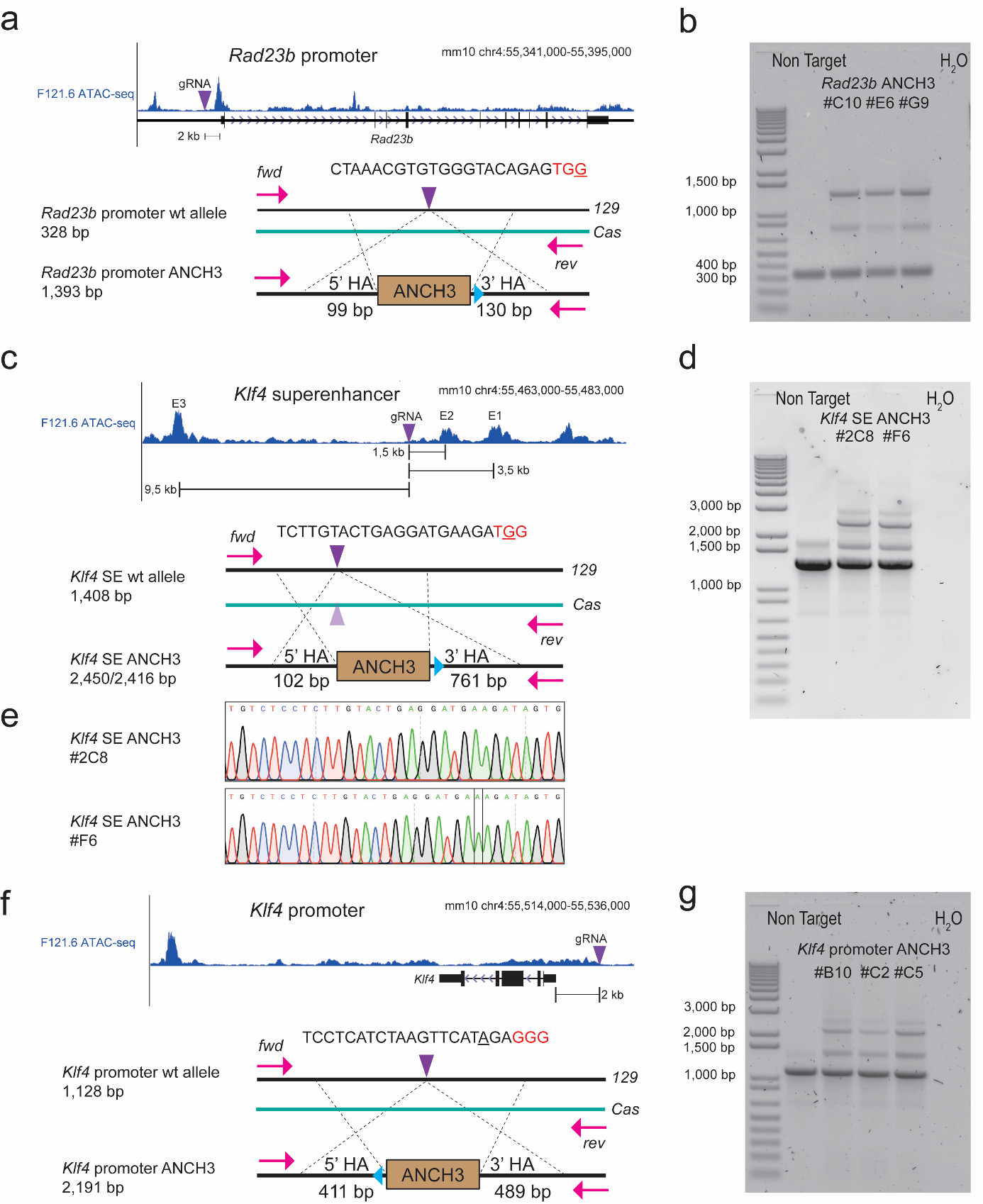
Fig. S1: Insertion of ANCH3 arrays at the cis-regulatory elements of the model locus.** (a,c,f) Overview of the insertion position of the ANCH3 arrays with respect to the transcription start site (TSS) of *Rad23b* (a), the *Klf4* enhancers (c) and TSS of *Klf4* (f) in the UCSC genome browser. F121.6 ATAC-sequencing data was downloaded from the 4DN Data Portal (4DNFI6HY3NE7)^1^. For each of the insertions, the genome editing strategy is depicted including the single guideRNA target sequence, the PAM sequence in red, the allele-specific SNP underlined and the length of the homology arms (HA) used for homologous recombination. On the left, the expected genotyping PCR product sizes for the wildtype and knock-in allele are shown. (b,d,g) Genotyping results of the heterozygous insertion of ANCH3 arrays at the desired genomic locations in different monoclonal cell lines. (e) Sanger sequencing tracks show that due the presence of a non-canonical PAM at the Castaneus (Cas) allele, a +1A insertion was observed at this allele of *Klf4* SE ANCH3 clone #F6.


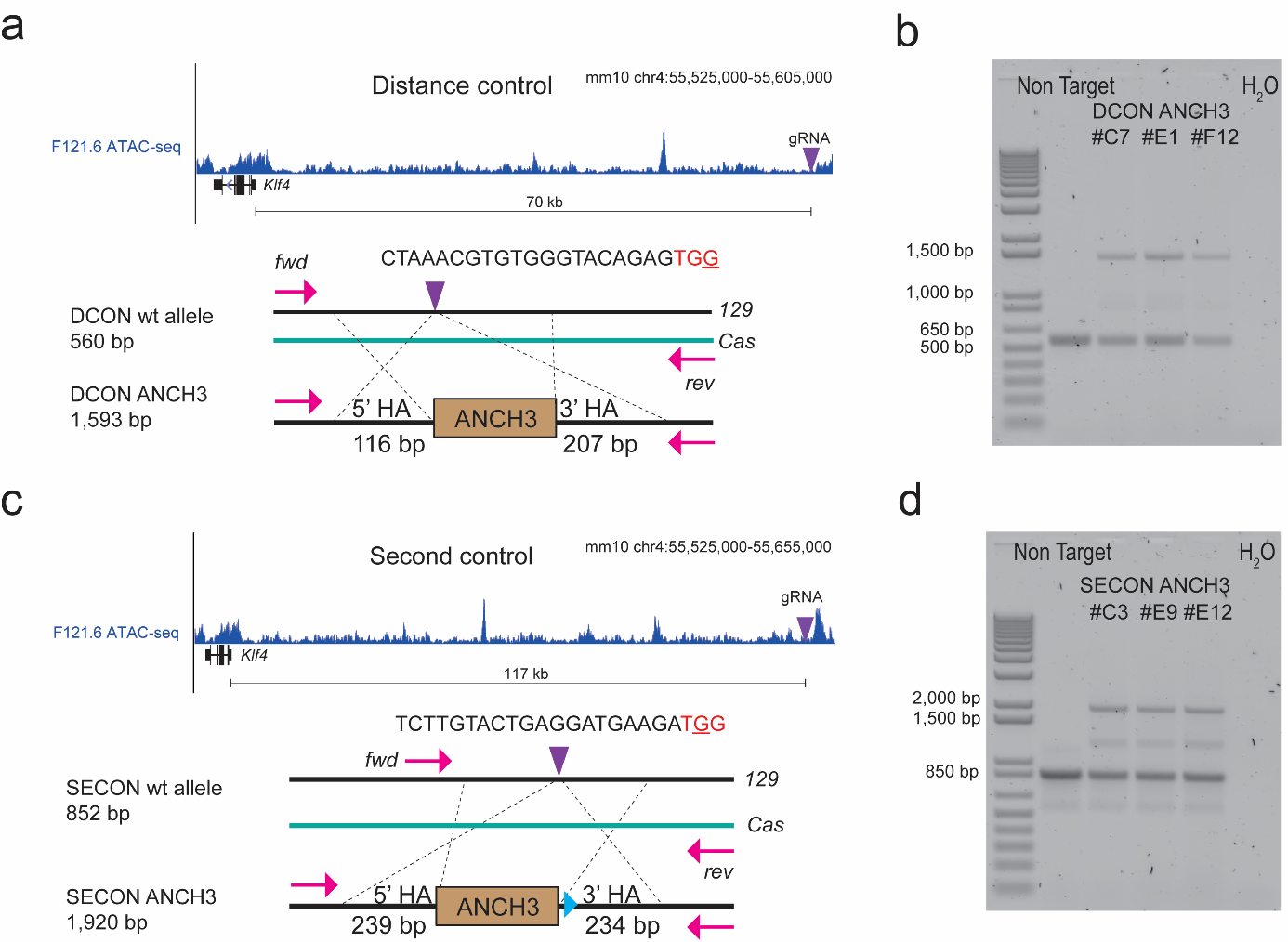


**Fig. S2: Insertion of ANCH3 arrays at the non-regulatory control regions of the model locus.** (a,c) Overview of the insertion position of the ANCH3 arrays with respect to the transcription start site (TSS) of *Klf4* in the UCSC genome browser. F121.6 ATAC-sequencing data was downloaded from the 4DN Data Portal (4DNFI6HY3NE7)^1^. For each of the insertions, the genome editing strategy is depicted including the single guideRNA target sequence, the PAM sequence in red, the allele-specific SNP underlined and the length of the homology arms (HA) used for homologous recombination. (b,d) On the left, the expected genotyping PCR product sizes for the wildtype and knock-in allele are shown. Genotyping results show the heterozygous insertion of ANCH3 arrays at the desired genomic locations in different monoclonal cell lines.


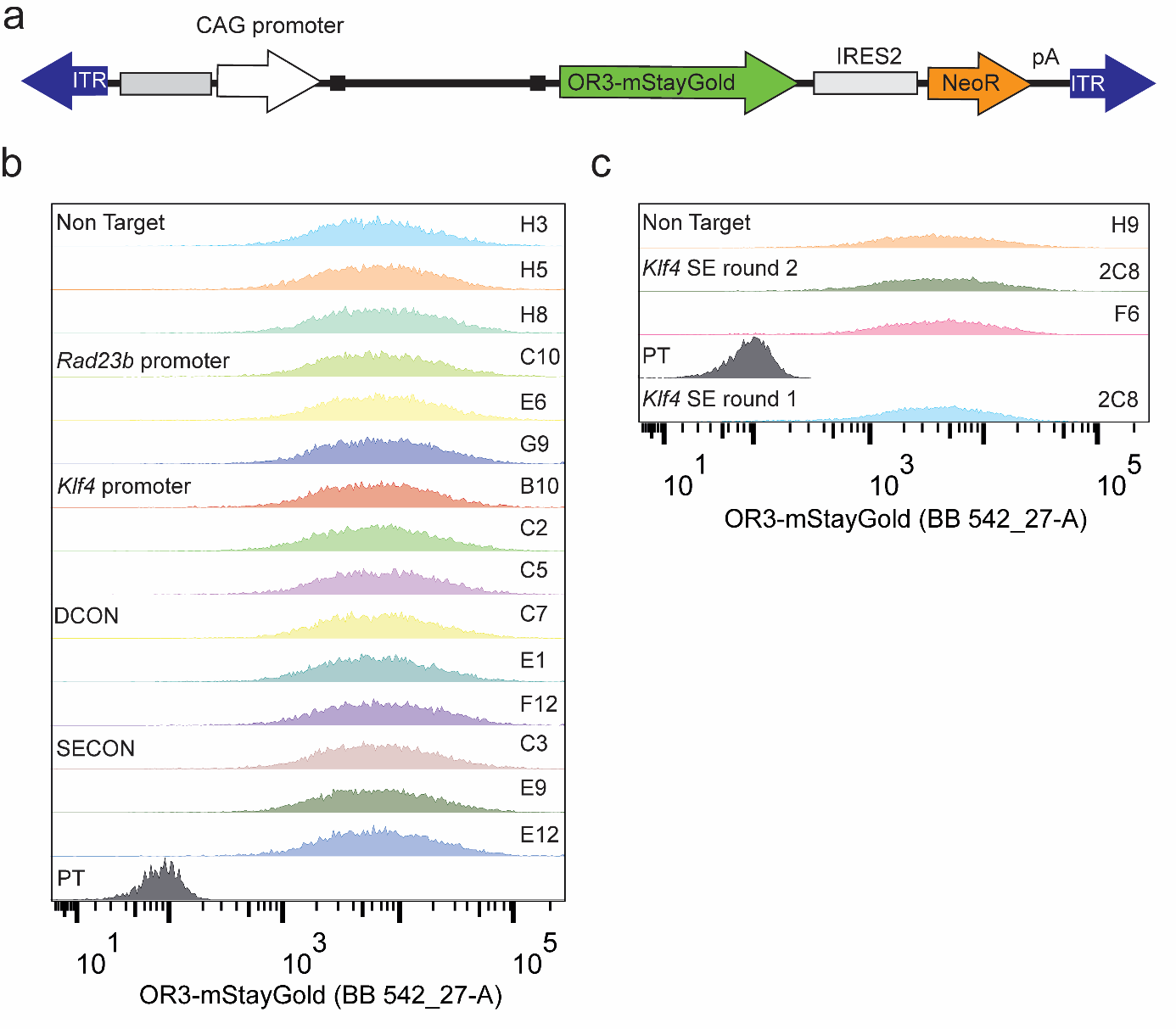


**Fig. S3: Transgenesis of ANCH3 edited clones with OR3-mStayGold expression cassettes.** (a) Schematic depiction of the OR3-mStayGold expression cassette used as cargo for PiggyBac mediated transposition. IRES2 = Internal ribosomal entry site, pA = poly A, ITR = inverted terminal repeat. (b,c) Flow cytometric analysis of stably selected transfection pools from two transfection rounds of ANCH3 clones, round 1 (b) and round 2 (c) 15 days and 13 days after transfection, respectively. PT = parental cell line.


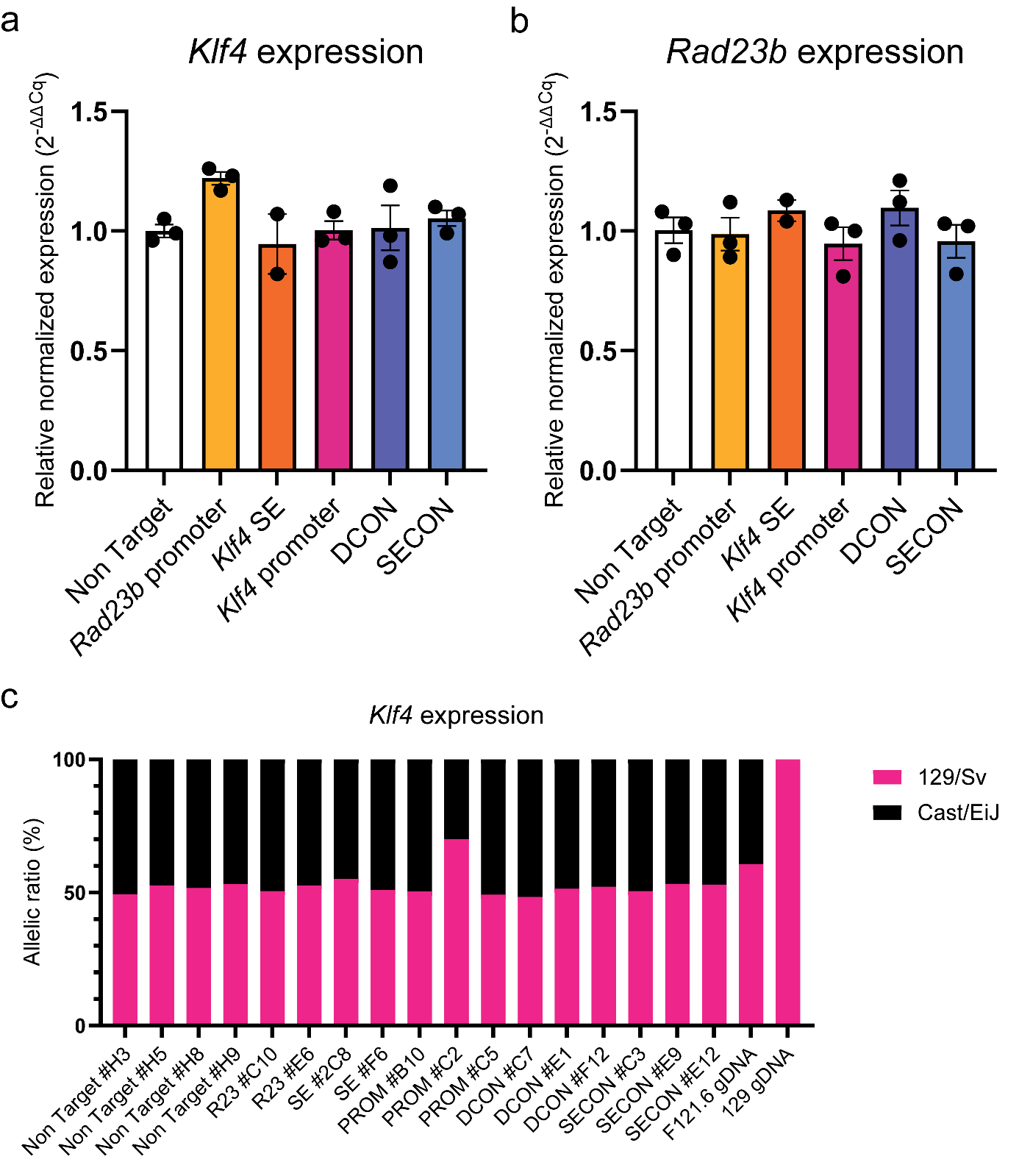


**Fig. S4: ANCHOR3 labeling does not perturb target gene expression.** (a,b) RT-qPCR analysis of *Klf4* (a) and *Rad23b* (b). Expression levels were normalized to the housekeeping genes *Sdha* and *Tbp* and are displayed relative to the values of non-targeted clones included on the same qPCR plate and derived from the same transfection round. Each dot represents the value of a clone. Bars indicate the mean value from three clones, except for the *Klf4* superenhancer, which includes two clones. Error bars indicate the standard error of the mean. (c) Pyrosequencing of *Klf4* shows the amount of 129 and Castaneus single nucleotide polymorphisms counted in the amplified complementary DNA of the indicated clones. Amplicons obtained from the genomic DNA of the parental F1-21.6 cell line and a 129X1/SvJ cell line were used as references.


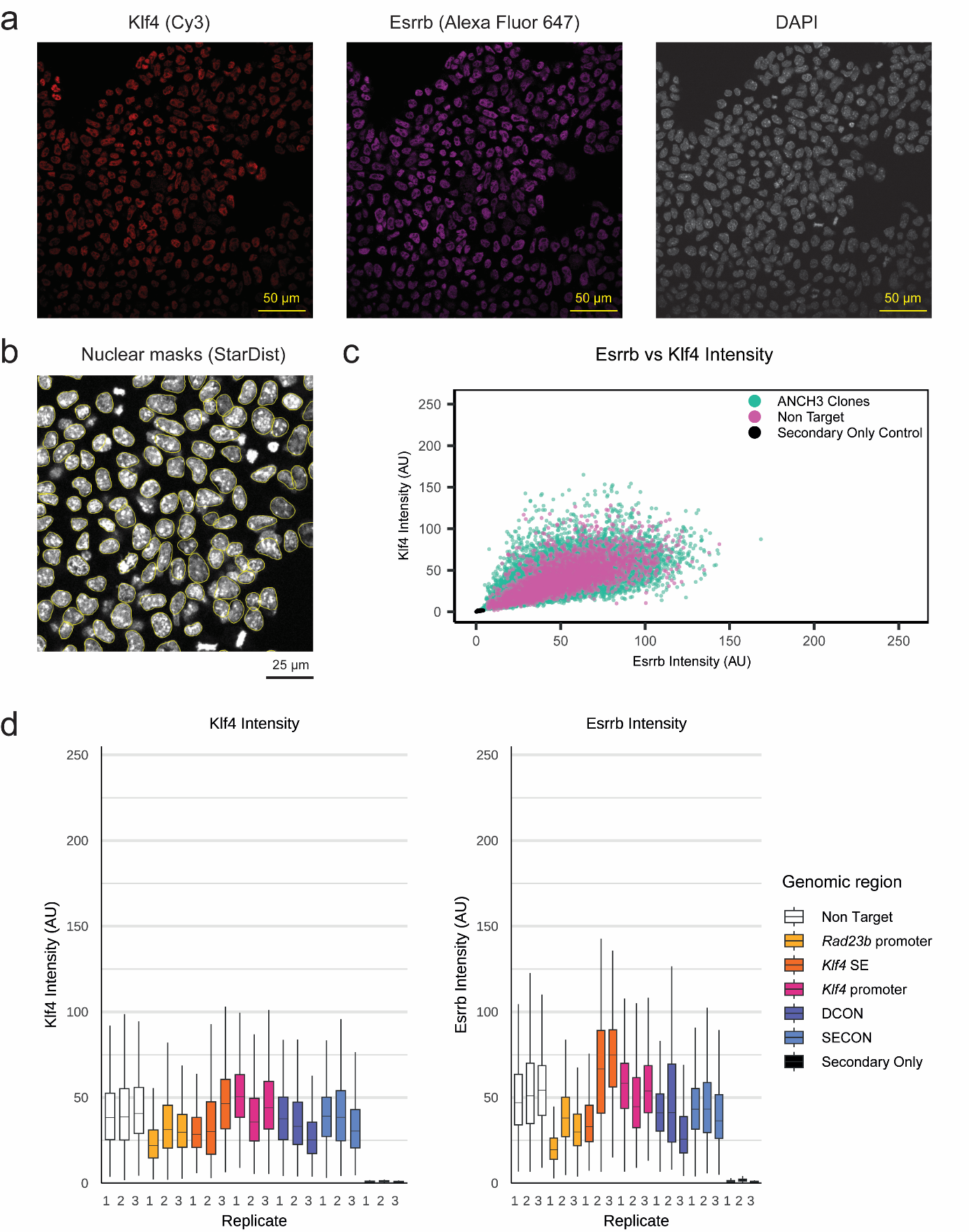


**Fig. S5: Quantification of Klf4 expression in naive mouse embryonic stem cells.** mESCs were grown in naïve conditions on laminin 511-LN coated imaging well chambers. (a) Representative images from an immunofluorescence staining performed on a non-targeted clone obtained by laser scanning confocal microscopy. (b) Result of nuclear segmentation obtained with StarDist. Detected nuclei are outlined in yellow. (c) Esrrb and Klf4 signal intensity for individual nuclei of ANCH3 edited clones (green) and non-targeted clones (magenta). The values obtained from the “secondary only control” are depicted in black. (d) The median Klf4 and Esrrb intensity signal in single nuclei of ANCH3 edited clones and non-targeted clones imaged in naive conditions. The boxes represent the interquartile range (IQR), while the whiskers extend to the most extreme values within 1.5 × IQR. The data is accumulated from imaging three technical

**Fig. S5 (continued):** replicates of a monoclonal cell line. The values obtained from the “secondary only control” are depicted in black.

**
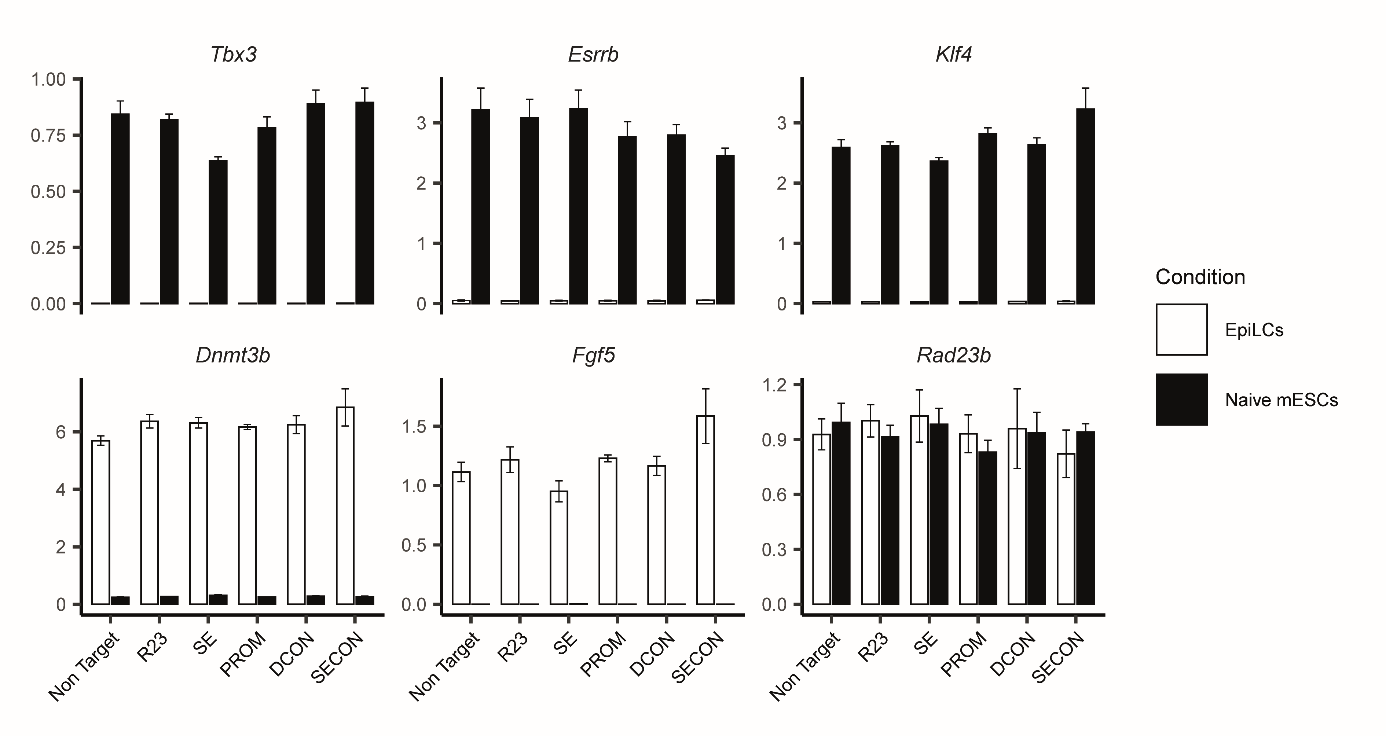
**

**Fig. S6: Quantification of gene expression in naive and primed conditions.** Expression of target genes *Klf4* and *Rad23b*, naive markers *Tbx3* and *Esrrb* and epiblast markers *Dnmt3b* and *Fgf5* in naive mESCs (black bars) and after differentiation to EpiLCs (white bars) measured with RT-qPCR. Values represent the average value obtained from three independent epiblast differentiations using a single ANCH3 edited OR3-mStayGold expressing clone. Error bars represent the standard error of the mean. All expression levels were normalized using *Sdha* and *Tbp* as reference genes. R23 = *Rad23b* promoter, SE = *Klf4* superenhancer, PROM = *Klf4* promoter, DCON = distance control region and SECON = second control region.

**
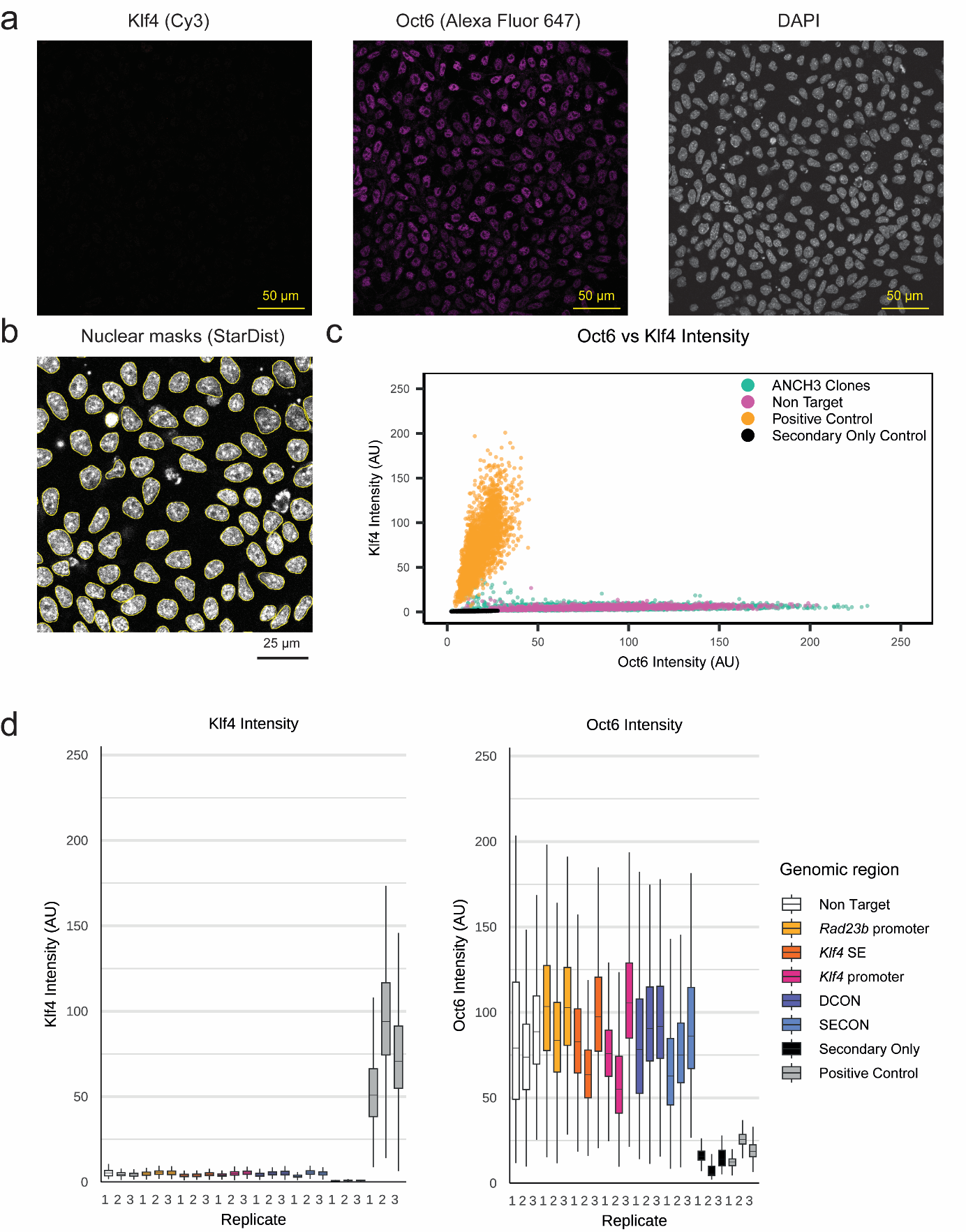
**

**Fig. S7: Quantification of Klf4 expression in epiblast-like cells.** (a) Representative images from an immunofluorescence staining performed on a non-targeted clone obtained by laser scanning confocal microscopy. (b) Result of nuclear segmentation obtained with StarDist. Detected nuclei are outlined in yellow. (c) Oct6 and Klf4 signal intensity for individual nuclei of ANCH3 edited clones (green) and non-targeted clones (magenta). The values obtained from the “secondary only control” are depicted in black and the values for the positive control (naive mESCs) are depicted in orange. (d) The median Klf4 and Oct6 signal intensity in single nuclei of ANCH3 edited clones and non-targeted clones imaged in primed conditions. The boxes represent the interquartile range (IQR), while the whiskers extend to the most extreme values within 1.5 × IQR. (*legend continues on next page*)

**Fig. S7 (continued):** The data is accumulated from imaging three technical replicates of a single clone. The values obtained from the “secondary only control” are depicted in black and the values obtained from the positive control for Klf4 (naive mouse embryonic stem cells) are depicted in grey.

**
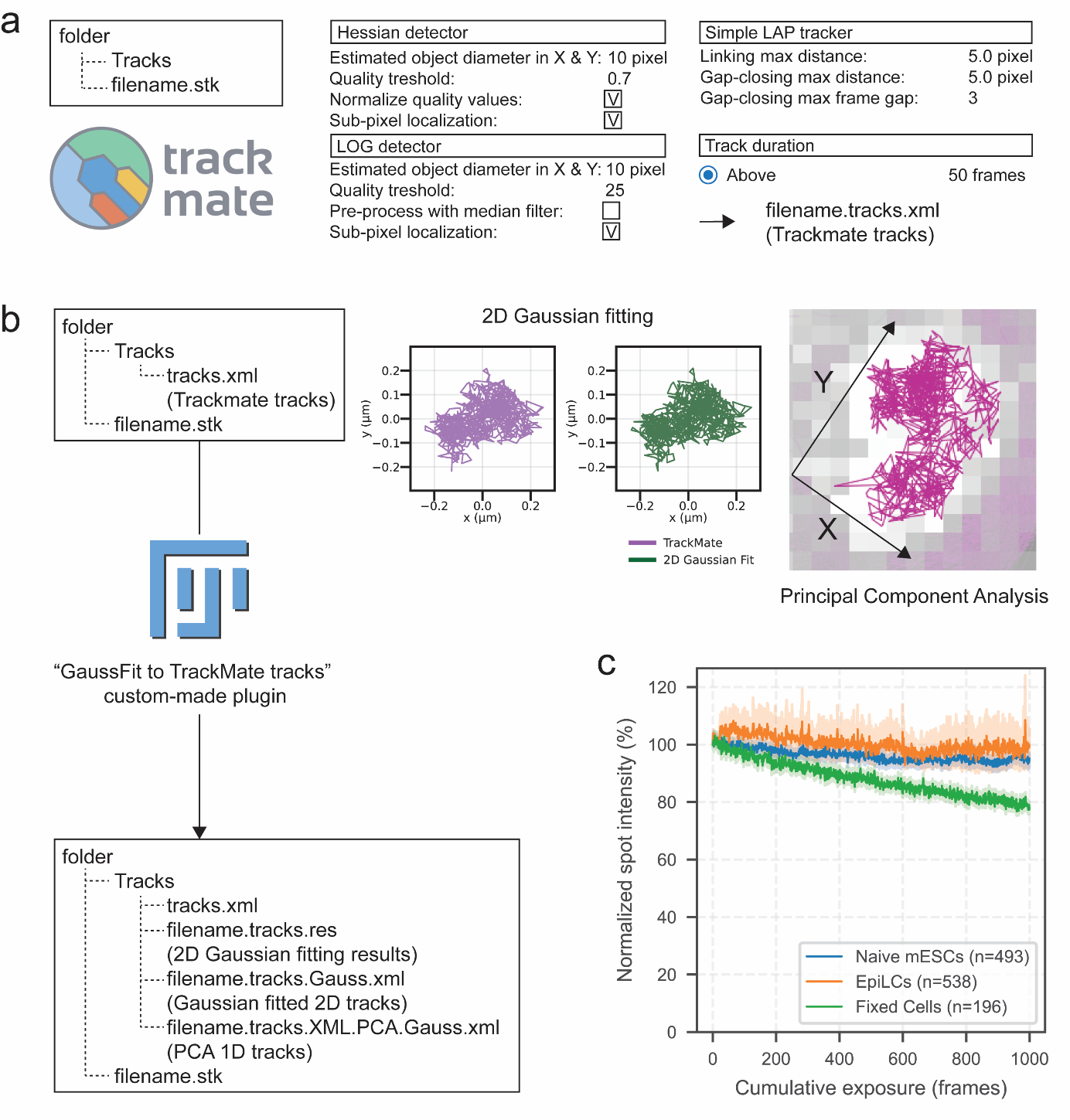
**

**Fig. S8: Tracking analysis pipeline.** (a) Spots are detected and tracked by TrackMate v7 with the indicated settings. (b) Tracks undergo Gaussian fitting and a principal component analysis with the “GaussFit to TrackMate tracks” plugin. A result of the Gaussian fitting is shown in the middle of the panel with an example track created by TrackMate (left) and after Gaussian Fitting (right). A Principal Component Analysis (PCA) is used to perform an alternative method of motion analysis (1D). As schematically depicted on the right. (c) The average spot intensity per frame is shown for naive mESCs, EpiLCs and fixed cells. Spot intensities are normalized to the intensity in the first frame.

**
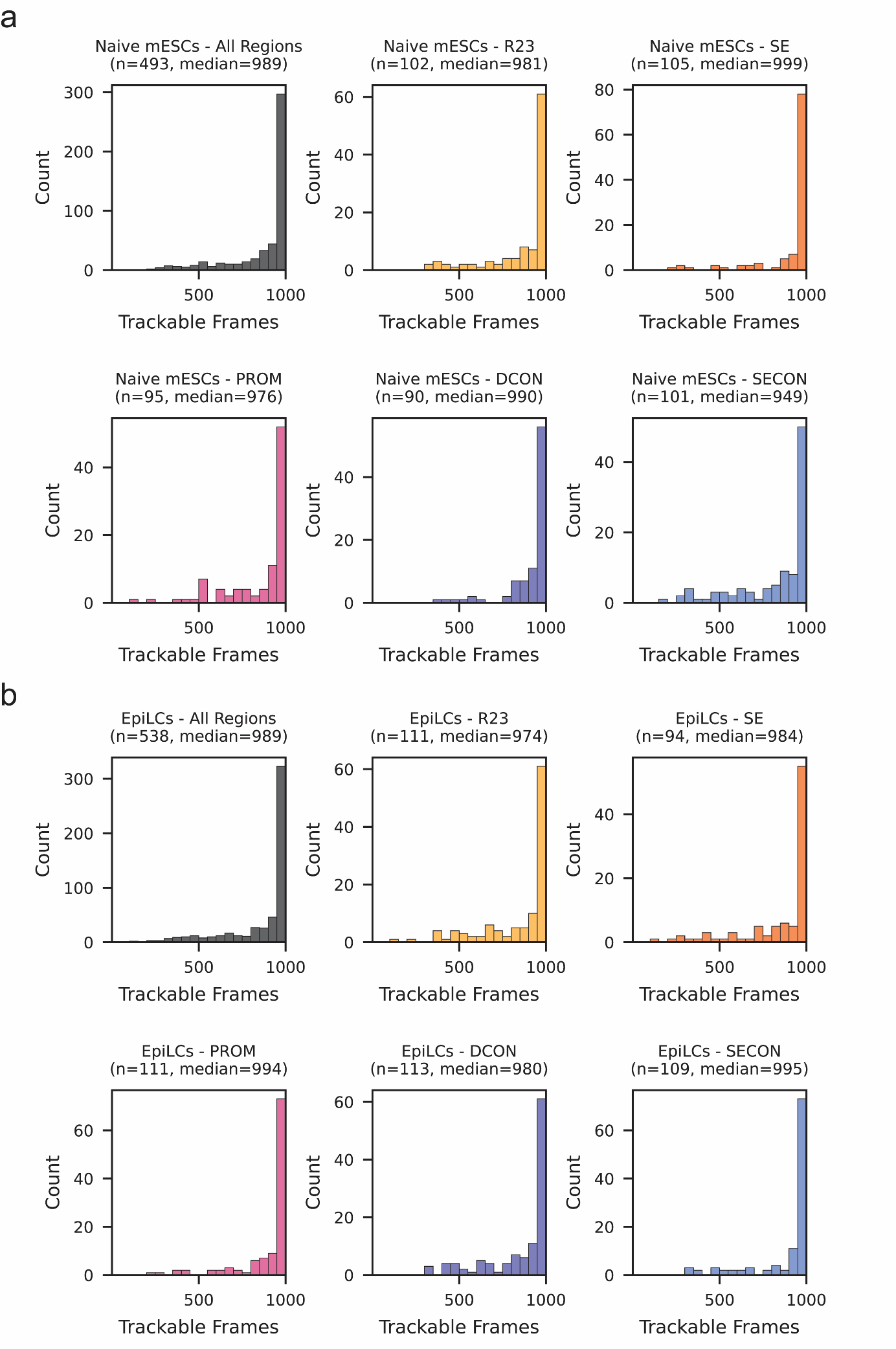
**

**Fig. S9 (Previous page): Trackable frames for OR3 spots across genomic regions.** Histograms of trackable frames stratified per genomic location in naive mouse embryonic stem cells (a) and epiblast-like cells (b). Count represents number of cells. R23 = *Rad23b* promoter, SE = *Klf4* superenhancer, PROM = *Klf4* promoter, DCON = distance control region and SECON = second control region.

**
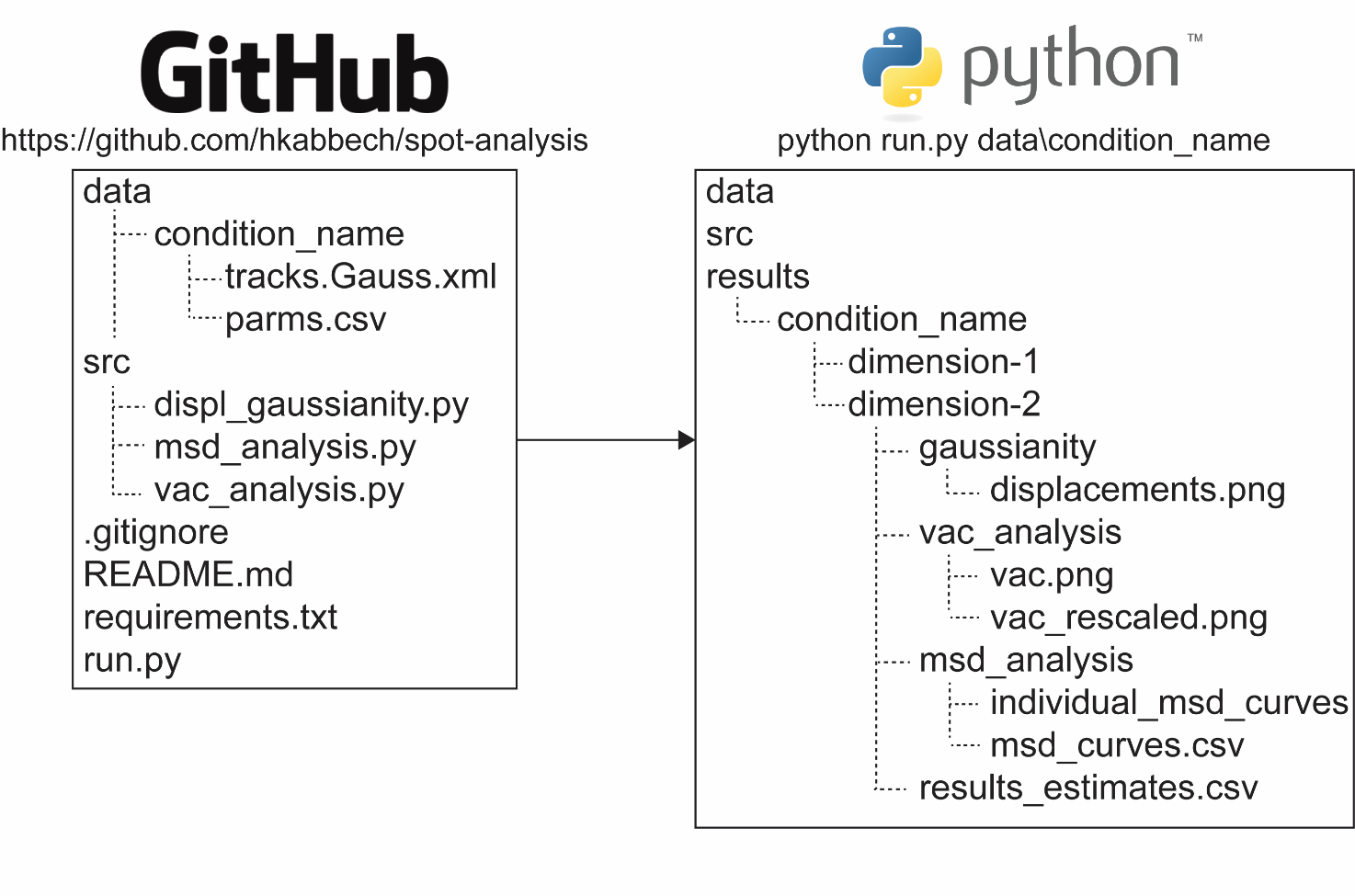
**

**Fig. S10: Tracking data analysis pipeline.** Tracking data analysis is performed with a Python script publicly available from GitHub. The script creates a results directory with the analysis output: a Gaussianity check, velocity autocorrelation curves and the ensemble time-averaged mean squared displacements curves. The estimated motion parameters (anomalous exponent and diffusion coefficient) obtained from fitting individual time averaged mean squared displacement curves are saved in the results_estimates.csv file.


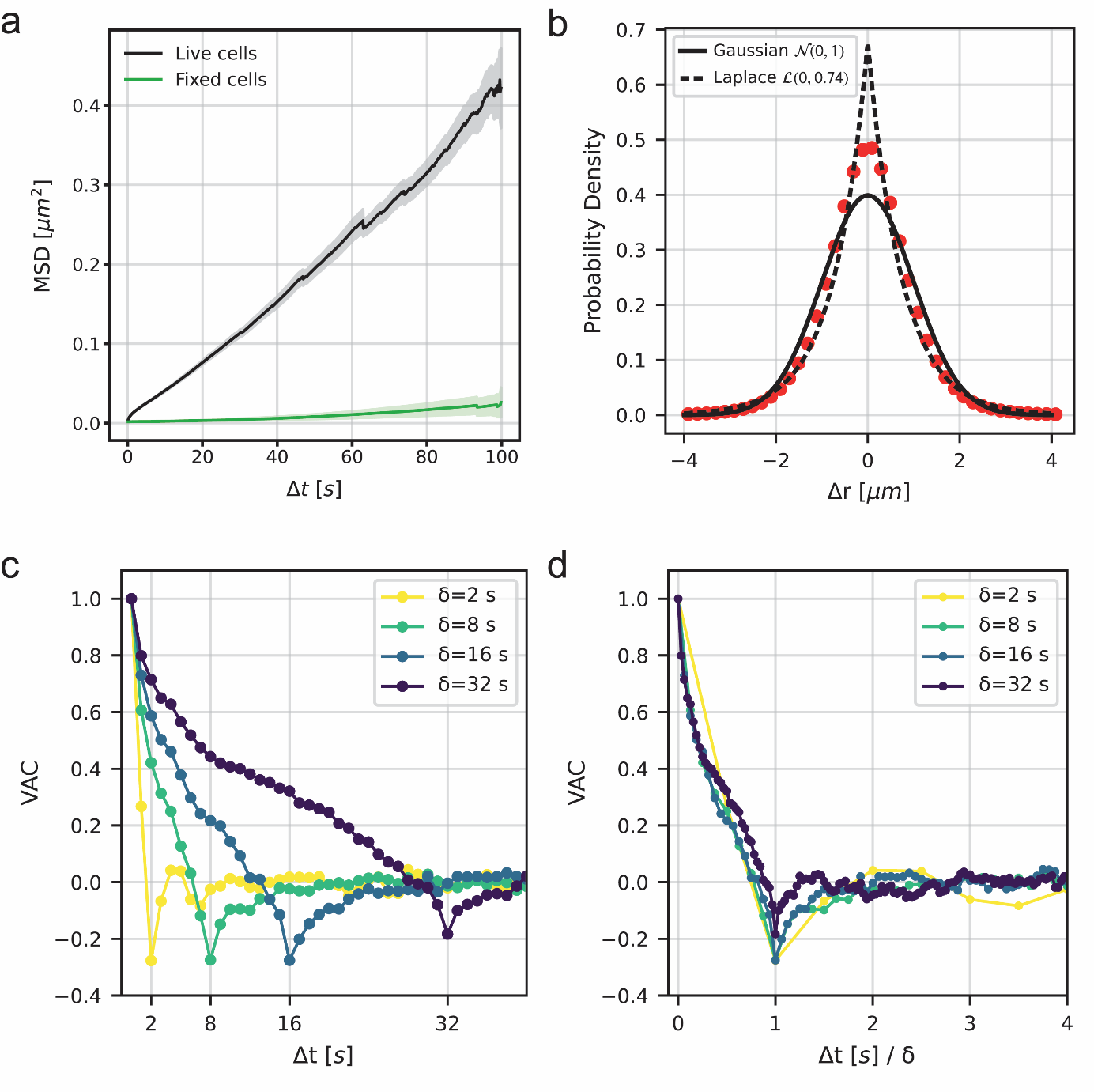


**Fig. S11: Characteristics of locus-wide chromatin dynamics.** (a) Ensemble time-averaged mean squared displacement (ETA-MSD) curves of all live cell imaged genomic regions (n = 1031) versus fixed cells (n = 196). The shading represents the standard error of the mean. (b) Probability density plot showing the distribution of displacements between consecutive frames (all data from live cells) together with fitted Gaussian and Laplacian distribution. (c) The velocity autocorrelation curves of all combined tracking data. (d) Normalized velocity autocorrelation curves for different time lags for all combined tracking data.

**
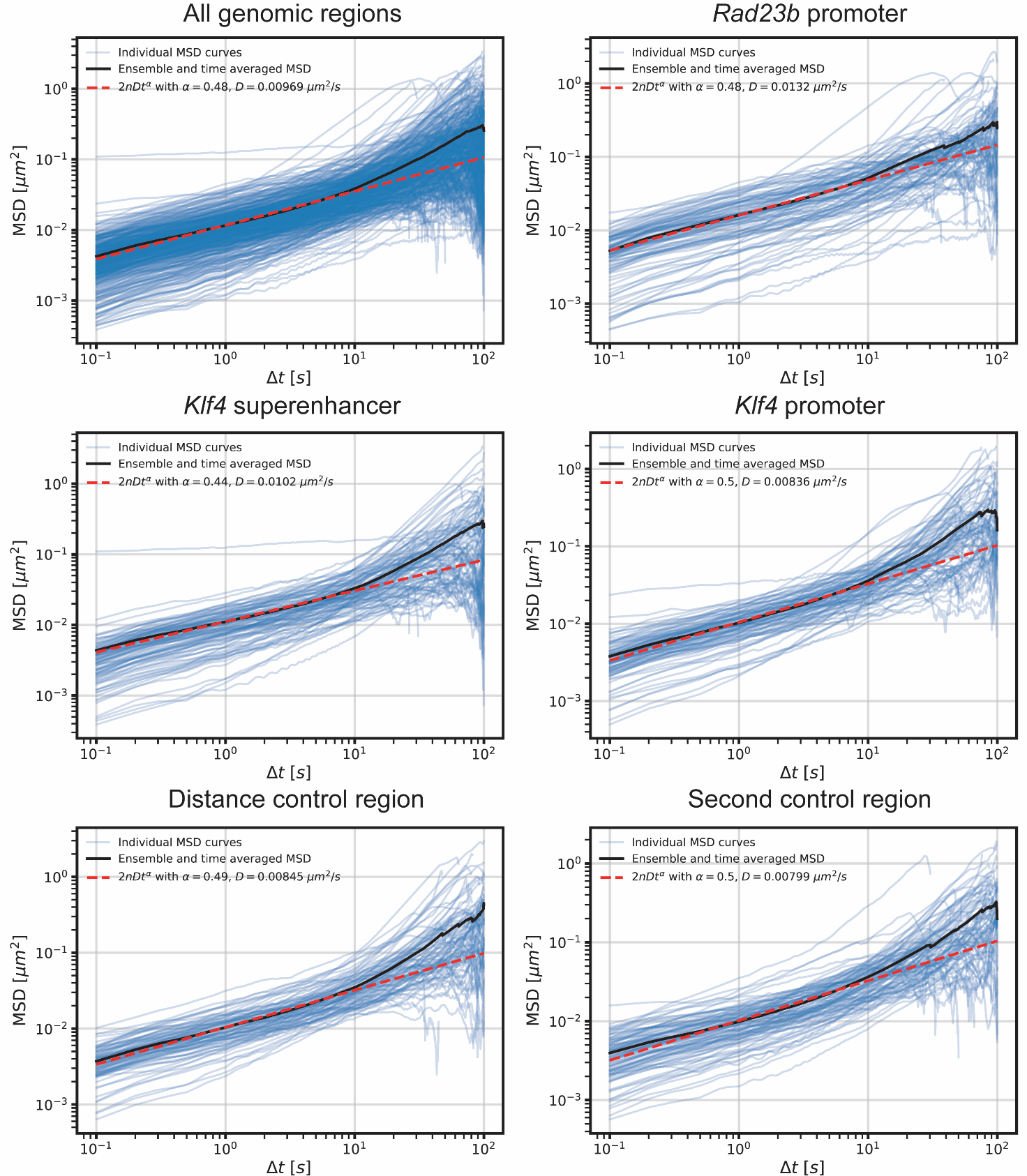
**

**Fig. S12: Mean squared displacement curves of the *Klf4* locus in naive mouse embryonic stem cells.** The collection of six subplots shows the ensemble time-averaged (ETA-MSD, black line) and individual time-averaged mean squared displacements (TA-MSD, blue lines) curves for all genomic regions in naive mouse embryonic stem cells, the *Rad23b* promoter, the *Klf4* superenhancer, the *Klf4* promoter, the distance control region and second control region in naive mouse embryonic stem cells. In the legend, the anomalous exponent and diffusion coefficients obtained from fitting the ETA-MSD curve are displayed (red dashed line). Tracking data was obtained from three biological replicates, except two biological replicates for the *Klf4* superenhancer in naive mESCs.


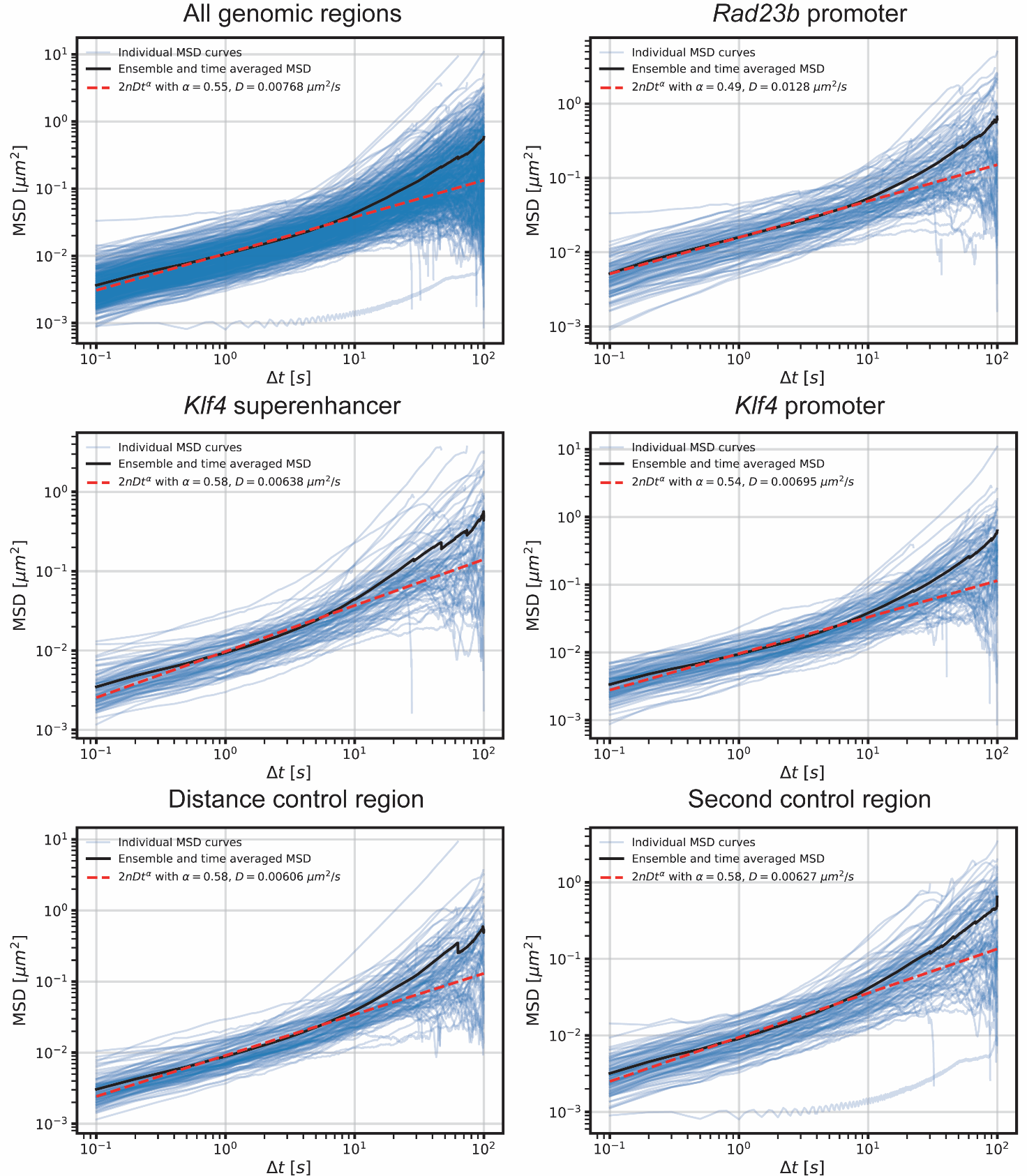


**Fig. S13: Mean squared displacement curves of the *Klf4* locus in epiblast-like cells.** The collection of six subplots shows the ensemble individual time-averaged (ETA-MSD, black line) and individual time-averaged mean squared displacements (TA-MSD, blue lines) curves for all genomic regions in epiblast-like cells, the *Rad23b* promoter, the *Klf4* superenhancer, the *Klf4* promoter, the distance control region and second control region in epiblast-like cells. In the legend, the anomalous exponent and diffusion coefficients obtained from fitting the ETA-MSD curve are displayed (red dashed line). Tracking data was obtained from three biological replicates, except two biological replicates for the *Klf4* superenhancer and distance control region in EpiLCs.


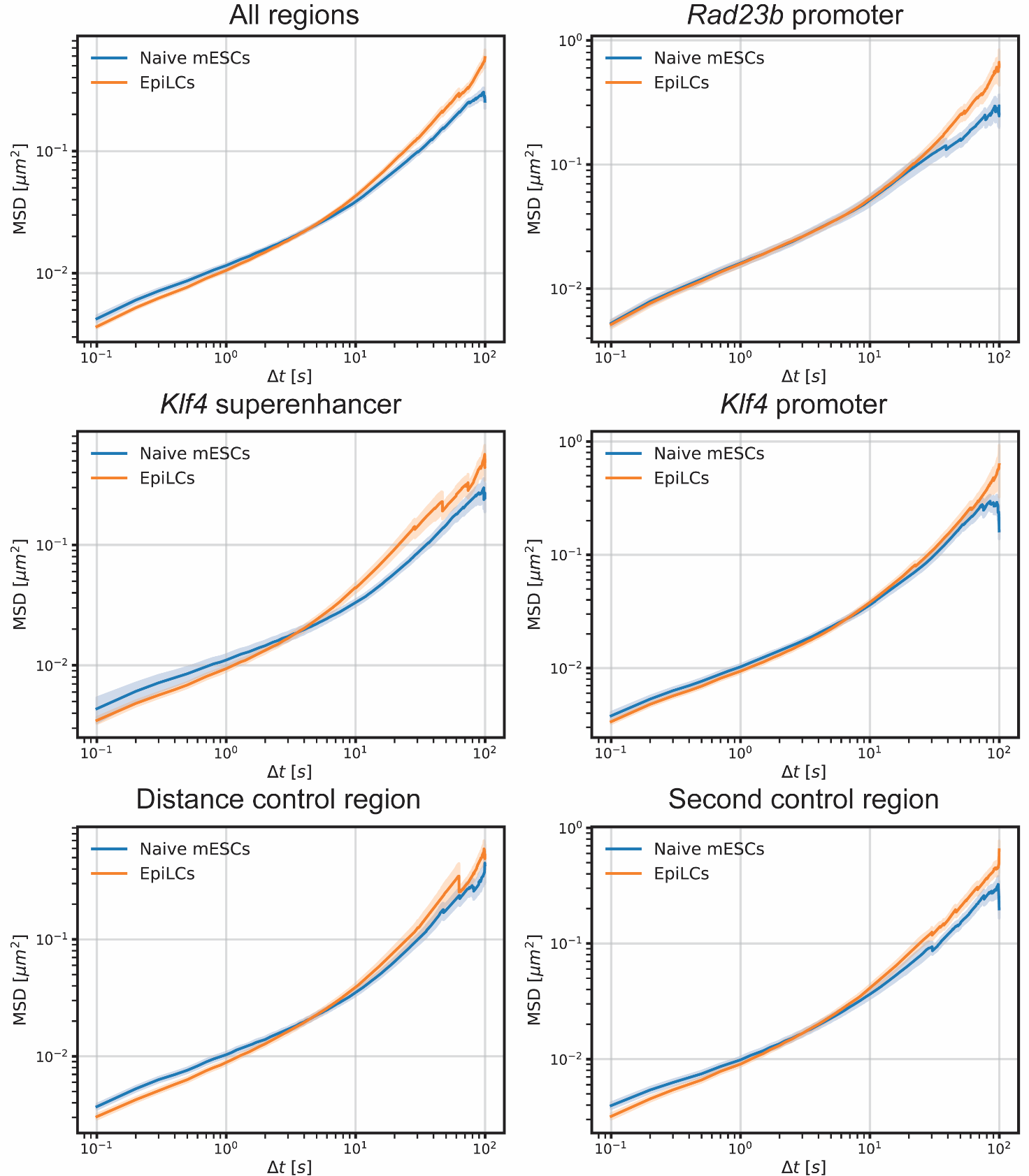
 **Fig. S14: Cell-type specific differences in chromatin motion.** The collection of six subplots show the ensemble individual time-averaged curves (ETA-MSD) for all regions, the *Rad23b* promoter (R23), the *Klf4* enhancer (SE), the *Klf4* promoter (PROM), the distance control region (DCON) and second control region (SECON) in naive mouse embryonic stem cells (blue) and epiblast-like cells (orange). The shading represents the standard error of the mean. The MSD curves were calculated from tracking data obtained from three biological replicates, except two biological replicates for DCON in EpiLCs and two for *Klf4* SE in naive mESCs and EpiLCs.


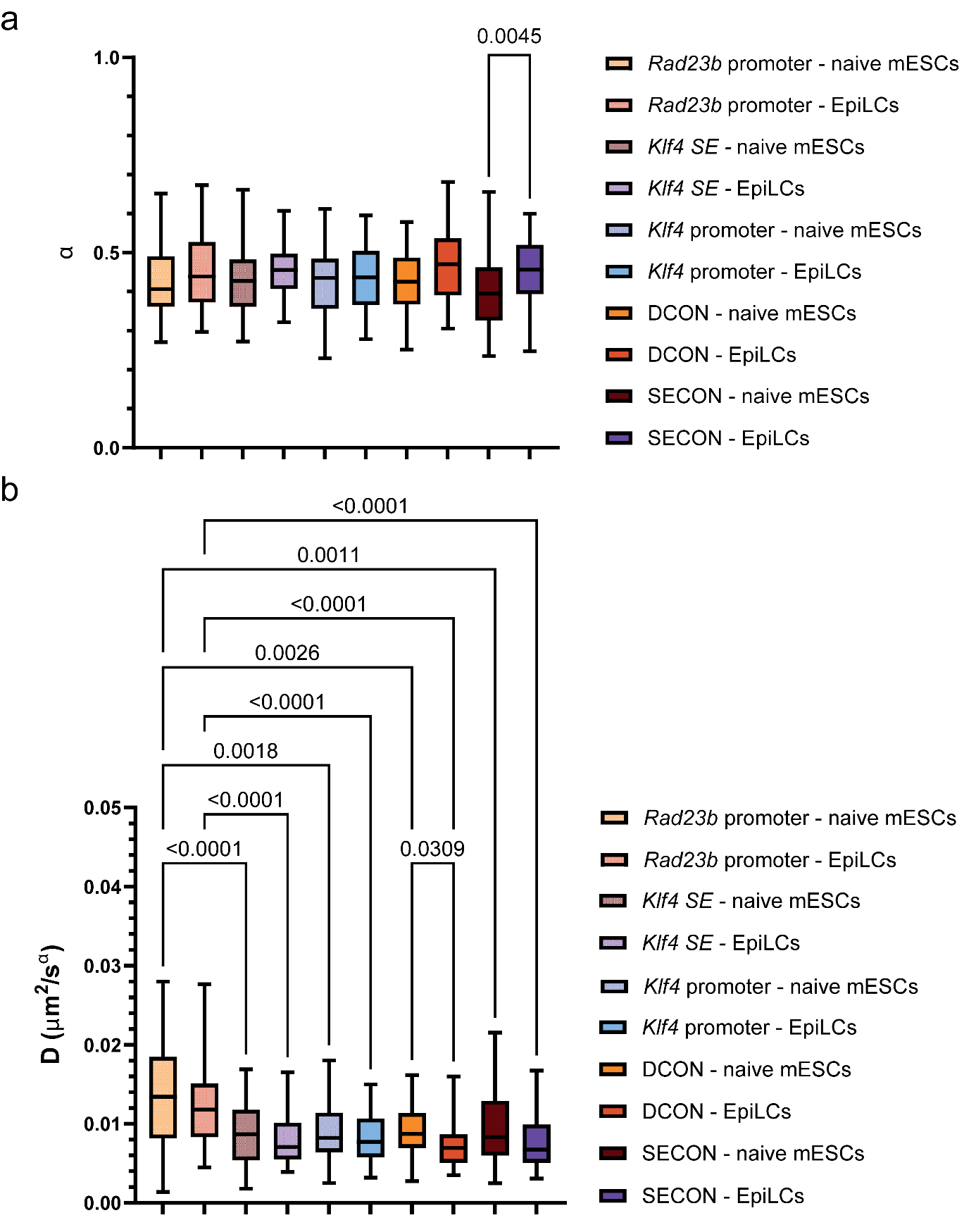


**Fig. S15: Alternative method of motion analysis.** XY coordinates of the 2D tracks underwent a principal component analysis leading to representation of each track in a new coordinate system. Displacements projected onto the axis perpendicular to the longest axis were used as input for the 1D MSD analysis. (a,b) Boxplots of the median anomalous exponents (a) and diffusion coefficients (b) in naive mouse embryonic stem cells and epiblast-like cells obtained from fitting the 1D tracking data of the *Rad23b* promoter, the *Klf4* superenhancer, the *Klf4* promoter, the distance control region (DCON) and second control region (SECON). The boxes cover data from the interquartile range (IQR) with whiskers extending to the 5-95^th^ percentile. Comparisons of the movement of genomic regions between cell types were made by performing the Kruskal-Wallis test followed by Dunn's multiple comparisons test. The motion parameters were obtained from fitting the time-averaged MSD curves of tracking data obtained from three biological replicates, except two biological replicates DCON in EpiLCs and two for *Klf4* SE in naive mESCs and EpiLCs.

**Supplementary sequence 1**

*Rad23b* promoter gBlocks Gene Fragment fasta sequence

>Rad23b_promoter

TGTTGTGGGATTGGATGTGAAGCCTCCTTAGAAGACAGCCACTGCTGATGGTTGTAGACATGGAAATCATGGGGGGAAGTGGCTAAACGTGTGGGTACATAGGGATAACAGGGTAATAGATCTATAACTTCGTATAGCATACATTATACGAAGTTATGAGTGGAGAGTAAATGTGATAAGACCTGACCCTGTTTTGGATGTGGTGGCTAGGCAGCAGGAGTCAGGGAAGAAAGGGACTTAAGGATGATGACGATGTTTGGATCTGAGTACCTAAGAGGTGGAATGTC

**Supplementary sequence 2**

SECON gBlocks Gene Fragment fasta sequence

>SECON

GGCCACTGGAGCCCACATTGTACGAGAAGAAGGGCCTCATTCAATGACCCCCTTTCATGTCGTTTTTTTTATCTTCAATATGGATGAGTCTTGGATTTGGAGTGGTCACAAACAAAAAGCACTTACTTTATGAATATAACGTGGGCAGTGGCATTATTTCAGTGGCTTTCCATAGTCCCGACTGACTTTTGGAATAGACTTATTGTAAATGAAGAGATAAGGCTTGCTTCACCCAATGTAGGGATAACAGGGTAATAGATCTTTGGTCCATTCATCCATTCATGCATTTGCACACTCGTTCATTCACTTGCTCATTCGTTTGCTCTTCGACTTCTTAAGTGTTTACTGAGCGTCACAATGTGTAGGGCACTTTAATCCAAGTGGGGTGTACTGTGGTAGGCAAATCGAGGTGGTTTCTGCATTCGTGAGTTTATGGGAAAGAAGAAGGAAGTCAAAAAACATCTGTGGCAAATTCTGTGGGGCAGCAGATGAGGTGCTGA

**Supplementary sequence 3**

PiggyBac cargo entry backbone fasta sequence

>PB_cargo_entry

TCAAAATGACGCATGATTATCTTTTACGTGACTTTTAAGATTTAACTCATACGATAATTATATTGTTATTTCGTGTTCTACTTACGTGATAACTTATTATATATATATTTTCTTGTTATAGATATCCTTCTCGAGCGACATTGATTATTGACTAGTTATTAATAGTAATCAATTACGGGGTCATTAGTTCATAGCCCATATATGGAGTTCCGCGTTACATAACTTACGGTAAATGGCCCGCCTGGCTGACCGCCCAACGACCCCCGCCCATTGACGTCAATAATGACGTATGTTCCCATAGTAACGCCAATAGGGACTTTCCATTGACGTCAATGGGTGGAGTATTTACGGTAAACTGCCCACTTGGCAGTACATCAAGTGTATCATATGCCAAGTACGCCCCCTATTGACGTCAATGACGGTAAATGGCCCGCCTGGCATTATGCCCAGTACATGACCTTATGGGACTTTCCTACTTGGCAGTACATCTACGTATTAGTCATCGCTATTACCATGGTCGAGGTGAGCCCCACGTTCTGCTTCACTCTCCCCATCTCCCCCCCCTCCCCACCCCCAATTTTGTATTTATTTATTTTTTAATTATTTTGTGCAGCGATGGGGGCGGGGGGGGGGGGGGGGCGCGCGCCRGGSGGGGSGGGGSGGGGSGRGGGGSGGGGSGGGGSGRGGSGGRGAGGTGCGGCGGCAGCCAATCAGAGCGGCGCGCTCCGAAAGTTTCCTTTTATGGCGAGGCGGCGGCGGCGGCGGCCCTATAAAAAGCGAAGCGCGCGGCGGGCGGGAGTCGCTGCGCGCTGCCTTCGCCCCGTGCCCCGCTCCGCCGCCGCCTCGCGCCGCCCGCCCCGGCTCTGACTGACCGCGTTACTCCCACAGGTGAGCGGGCGGGACGGCCCTTCTCCTCCGGGCTGTAATTAGCGCTTGGTTTAATGACGGCTTGTTTCTTTTCTGTGGCTGCGTGAAAGCCTTGAGGGGCTCCGGGAGGGCCCTTTGTGCGGGGGGAGCGGCTCGGGGGGTGCGTGCGTGTGTGTGTGCGTGGGGAGCGCCGCGTGCGGCTCCGCGCTGCCCGGCGGCTGTGAGCGCTGCGGGCGCGGCGCGGGGCTTTGTGCGCTCCGCAGTGTGCGCGAGGGGAGCGCGGCCGGGGGCGGTGCCCCGCGGTGCGGGGGGGGCTGCGAGGGGAACAAAGGCTGCGTGCGGGGTGTGTGCGTGGGGGGGTGAGCAGGGGGTGTGGGCGCGTCGGTCGGGCTGCAACCCCCCCTGCACCCCCCTCCCCGAGTTGCTGAGCACGGCCCGGCTTCGGGTGCGGGGCTCCGTACGGGGCGTGGCGCGGGGCTCGCCGTGCCGGGCGGGGGGTGGCGGCAGGTGGGGGTGCCGGGCGGGGCGGGGCCGCCTCGGGCCGGGGAGGGCTCGGGGGAGGGGCGCGGCGGCCCCCGGAGCGCCGGCGGCTGTCGAGGCGCGGCGAGCCGCAGCCATTGCCTTTTATGGTAATCGTGCGAGAGGGCGCAGGGACTTCCTTTGTCCCAAATCTGTGCGGAGCCGAAATCTGGGAGGCGCCGCCGCACCCCCTCTAGCGGGCGCGGGGCGAAGCGGTGCGGCGCCGGCAGGAAGGAAATGGGCGGGGAGGGCCTTCGTGCGTCGCCGCGCCGCCGTCCCCTTCTCCCTCTCCAGCCTCGGGGCTGTCCGCGGGGGGACGGCTGCCTTCGGGGGGGACGGGGCAGGGCGGGGTTCGGCTTCTGGCGTGTGACCGGCGGCTCTAGAGCCTCTGCTAACCATGTTCATGCCTTCTTCTTTTTCCTACAGCTCCTGGGCAACGTGCTGGTTATTGTGCTGTCTCATCATTTTGGCAAAGAATTGCTCGAGGGCGGCCGCGCCCCTCTCCCTCCCCCCCCCCTAACGTTACTGGCCGAAGCCGCTTGGAATAAGGCCGGTGTGCGTTTGTCTATATGTTATTTTCCACCATATTGCCGTCTTTTGGCAATGTGAGGGCCCGGAAACCTGGCCCTGTCTTCTTGACGAGCATTCCTAGGGGTCTTTCCCCTCTCGCCAAAGGAATGCAAGGTCTGTTGAATGTCGTGAAGGAAGCAGTTCCTCTGGAAGCTTCTTGAAGACAAACAACGTCTGTAGCGACCCTTTGCAGGCAGCGGAACCCCCCACCTGGCGACAGGTGCCTCTGCGGCCAAAAGCCACGTGTATAAGATACACCTGCAAAGGCGGCACAACCCCAGTGCCACGTTGTGAGTTGGATAGTTGTGGAAAGAGTCAAATGGCTCTCCTCAAGCGTATTCAACAAGGGGCTGAAGGATGCCCAGAAGGTACCCCATTGTATGGGATCTGATCTGGGGCCTCGGTGCACATGCTTTACATGTGTTTAGTCGAGGTTAAAAAAACGTCTAGGCCCCCCGAACCACGGGGACGTGGTTTTCCTTTGAAAAACACGATGATAATATGGCCACAACCATGGCGTCCGGAATGATTGAACAAGATGGATTGCACGCAGGTTCTCCGGCCGCTTGGGTGGAGAGGCTATTCGGCTATGACTGGGCACAACAGACAATCGGCTGCTCTGATGCCGCCGTGTTCCGGCTGTCAGCGCAGGGGCGCCCGGTTCTTTTTGTCAAGACCGACCTGTCCGGTGCCCTGAATGAACTGCAGGACGAGGCAGCGCGGCTATCGTGGCTGGCCGCGACGGGCGTTCCTTGCGCAGCTGTGCTCGACGTTGTCACTGAAGCGGGAAGGGACTGGCTGCTATTGGGCGAAGTGCCGGGGCAGGATCTCCTGTCATCTCACCTTGCTCCTGCCGAGAAAGTATCCATCATGGCTGATGCAATGCGGCGGCTGCATACGCTTGATCCGGCTACCTGCCCATTCGACCACCAAGCGAAACATCGCATCGAGCGAGCACGTACTCGGATGGAAGCCGGTCTTGTCGATCAGGATGATCTGGACGAAGAGCATCAGGGGCTCGCGCCAGCCGAACTGTTCGCCAGGCTCAAGGCGCGCATGCCCGACGGCGAGGATCTCGTCGTGACCCATGGCGATGCCTGCTTGCCGAATATCATGGTGGAAAATGGCCGCTTTTCTGGATTCATCGACTGTGGCCGGCTGGGTGTGGCGGACCGCTATCAGGACATAGCGTTGGCTACCCGTGATATTGCTGAAGAGCTTGGCGGCGAATGGGCTGACCGCTTCCTCGTGCTTTACGGTATCGCCGCTCCCGATTCGCAGCGCATCGCCTTCTATCGCCTTCTTGACGAGTTCTTCTGAGTCGACAATCAACCTCTGGATTACAAAATTTGTGAAAGATTGACTGGTATTCTTAACTATGTTGCTCCTTTTACGCTATGTGGATACGCTGCTTTAATGCCTTTGTATCATGCGTTAACTAAACTTGTTTATTGCAGCTTATAATGGTTACAAATAAAGCAATAGCATCACAAATTTCACAAATAAAGCATTTTTTTCACTGCATTCTAGTTGTGGTTTGTCCAAACTCATCAATGTATCTTAGGAATTCGATAAAAGTTTTGTTACTTTATAGAAGAAATTTTGAGTTTTTGTTTTTTTTTAATAAATAAATAAACATAAATAAATTGTTTGTTGAATTTATTATTAGTATGTAAGTGTAAATATAATAAAACTTAATATCTATTCAAATTAATAAATAAACCTCGATATACAGACCGATAAAACACATGCGTCAATTTTACGCATGATTATCTTTAACGTACGTCACAATATGATTATCTTTCTAGGGTTAATCTAGCTGCGGCGCGCCGGTACCCAATTCGCCCTATAGTGAGTCGTATTACGCGCGCTCACTGGCCGTCGTTTTACAACGTCGTGACTGGGAAAACCCTGGCGTTACCCAACTTAATCGCCTTGCAGCACATCCCCCTTTCGCCAGCTGGCGTAATAGCGAAGAGGCCCGCACCGATCGCCCTTCCCAACAGTTGCGCAGCCTGAATGGCGAATGGGACGCGCCCTGTAGCGGCGCATTAAGCGCGGCGGGTGTGGTGGTTACGCGCAGCGTGACCGCTACACTTGCCAGCGCCCTAGCGCCCGCTCCTTTCGCTTTCTTCCCTTCCTTTCTCGCCACGTTCGCCGGCTTTCCCCGTCAAGCTCTAAATCGGGGGCTCCCTTTAGGGTTCCGATTTAGTGCTTTACGGCACCTCGACCCCAAAAAACTTGATTAGGGTGATGGTTCACGTAGTGGGCCATCGCCCTGATAGACGGTTTTTCGCCCTTTGACGTTGGAGTCCACGTTCTTTAATAGTGGACTCTTGTTCCAAACTGGAACAACACTCAACCCTATCTCGGTCTATTCTTTTGATTTATAAGGGATTTTGCCGATTTCGGCCTATTGGTTAAAAAATGAGCTGATTTAACAAAAATTTAACGCGAATTTTAACAAAATATTAACGCTTACAATTTAGGTGGCACTTTTCGGGGAAATGTGCGCGGAACCCCTATTTGTTTATTTTTCTAAATACATTCAAATATGTATCCGCTCATGAGACAATAACCCTGATAAATGCTTCAATAATATTGAAAAAGGAAGAGTATGAGTATTCAACATTTCCGTGTCGCCCTTATTCCCTTTTTTGCGGCATTTTGCCTTCCTGTTTTTGCTCACCCAGAAACGCTGGTGAAAGTAAAAGATGCTGAAGATCAGTTGGGTGCACGAGTGGGTTACATCGAACTGGATCTCAACAGCGGTAAGATCCTTGAGAGTTTTCGCCCCGAAGAACGTTTTCCAATGATGAGCACTTTTAAAGTTCTGCTATGTGGCGCGGTATTATCCCGTATTGACGCCGGGCAAGAGCAACTCGGTCGCCGCATACACTATTCTCAGAATGACTTGGTTGAGTACTCACCAGTCACAGAAAAGCATCTTACGGATGGCATGACAGTAAGAGAATTATGCAGTGCTGCCATAACCATGAGTGATAACACTGCGGCCAACTTACTTCTGACAACGATCGGAGGACCGAAGGAGCTAACCGCTTTTTTGCACAACATGGGGGATCATGTAACTCGCCTTGATCGTTGGGAACCGGAGCTGAATGAAGCCATACCAAACGACGAGCGTGACACCACGATGCCTGTAGCAATGGCAACAACGTTGCGCAAACTATTAACTGGCGAACTACTTACTCTAGCTTCCCGGCAACAATTAATAGACTGGATGGAGGCGGATAAAGTTGCAGGACCACTTCTGCGCTCGGCCCTTCCGGCTGGCTGGTTTATTGCTGATAAATCTGGAGCCGGTGAGCGTGGGTCTCGCGGTATCATTGCAGCACTGGGGCCAGATGGTAAGCCCTCCCGTATCGTAGTTATCTACACGACGGGGAGTCAGGCAACTATGGATGAACGAAATAGACAGATCGCTGAGATAGGTGCCTCACTGATTAAGCATTGGTAACTGTCAGACCAAGTTTACTCATATATACTTTAGATTGATTTAAAACTTCATTTTTAATTTAAAAGGATCTAGGTGAAGATCCTTTTTGATAATCTCATGACCAAAATCCCTTAACGTGAGTTTTCGTTCCACTGAGCGTCAGACCCCGTAGAAAAGATCAAAGGATCTTCTTGAGATCCTTTTTTTCTGCGCGTAATCTGCTGCTTGCAAACAAAAAAACCACCGCTACCAGCGGTGGTTTGTTTGCCGGATCAAGAGCTACCAACTCTTTTTCCGAAGGTAACTGGCTTCAGCAGAGCGCAGATACCAAATACTGTTCTTCTAGTGTAGCCGTAGTTAGGCCACCACTTCAAGAACTCTGTAGCACCGCCTACATACCTCGCTCTGCTAATCCTGTTACCAGTGGCTGCTGCCAGTGGCGATAAGTCGTGTCTTACCGGGTTGGACTCAAGACGATAGTTACCGGATAAGGCGCAGCGGTCGGGCTGAACGGGGGGTTCGTGCACACAGCCCAGCTTGGAGCGAACGACCTACACCGAACTGAGATACCTACAGCGTGAGCTATGAGAAAGCGCCACGCTTCCCGAAGGGAGAAAGGCGGACAGGTATCCGGTAAGCGGCAGGGTCGGAACAGGAGAGCGCACGAGGGAGCTTCCAGGGGGAAACGCCTGGTATCTTTATAGTCCTGTCGGGTTTCGCCACCTCTGACTTGAGCGTCGATTTTTGTGATGCTCGTCAGGGGGGCGGAGCCTATGGAAAAACGCCAGCAACGCGGCCTTTTTACGGTTCCTGGCCTTTTGCTGGCCTTTTGCTCACATGTTCTTTCCTGCGTTATCCCCTGATTCTGTGGATAACCGTATTACCGCCTTTGAGTGAGCTGATACCGCTCGCCGCAGCCGAACGACCGAGCGCAGCGAGTCAGTGAGCGAGGAAGCGGAAGAGCGCCCAATACGCAAACCGCCTCTCCCCGCGCGTTGGCCGATTCATTAATGCAGCTGGCACGACAGGTTTCCCGACTGGAAAGCGGGCAGTGAGCGCAACGCAATTAATGTGAGTTAGCTCACTCATTAGGCACCCCAGGCTTTACACTTTATGCTTCCGGCTCGTATGTTGTGTGGAATTGTGAGCGGATAACAATTTCACACAGGAAACAGCTATGACCATGATTACGCCAAGCGCGCAATTAACCCTCACTAAAGGGAACAAAAGCTGGAGCTCCACCGCGGGGCGCGCCGCAGCTAGATTAACCCTAGAAAGATAGTCTGCGTAAAATTGACGCATGCATTCTTGAAATATTGCTCTCTCTTTCTAAATAGCGCGAATCCGTCGCTGTGCATTTAGGACATCTCAGTCGCCGCTTGGAGCTCCCGTGAGGCGTGCTTGTCAATGCGGTAAGTGTCACTGATTTTGAACTATAACAACCGCGTGAG

**References**

1. Rivera-Mulia, J. C. *et al.* Allele-specific control of replication timing and genome organization during development. *Genome Res.* **28**, 800–811 (2018).
